# Improved modelling for vibrational energies of diatomic molecules using the generalized fractional derivative

**DOI:** 10.1038/s41598-026-39091-5

**Published:** 2026-04-09

**Authors:** E. M. Khokha, M. Abu-Shady, E. Omugbe, N. S. Sweed

**Affiliations:** 1 Faculty of Computer Science and Engineering, King Salman International University (KSIU), El Tur, South Sinai 46511 Egypt; 2 Department of Mathematics and Computer Science, Faculty of Science, Menoufia University, Shibin El Kom, 32511 Egypt; 3 Department of Physics and Engineering Physics, Faculty of Science, Obafemi Awolowo University, Ile-Ife, Osun State Nigeria; 4 Department of Basic Science, Modern Academy for Engineering and Technology, Cairo, 11439 Egypt

**Keywords:** Radial Schrödinger Equation, Generalized Fractional Derivatives, Nikiforov–Uvarov Method, Morse Potential, Diatomic Molecules, Chemistry, Mathematics and computing, Physics

## Abstract

By using the radial Schrödinger equation with the Morse potential in the context of the generalized fractional derivative (GFD), this work provides an important improvement in modelling the vibrational energy spectrum of diatomic molecules. We have used the generalized fractional Nikiforov-Uvarov (GFNU) method to derive an analytical solution for the energy eigenvalues in *D*-dimensional space by applying the Pekeris-type approximation to the centrifugal term. The proposed model is thoroughly examined across many electronic states, using a diverse set of twenty-two diatomic molecules, including astrophysically important species like SiO$^+$ and TaO, as well as CO, Na$_2$, and AlH. The potential energy curves for the selected diatomic molecules have been produced using the Morse potential with the help of molecular constants. Furthermore, the pure vibrational energy levels for several diatomic molecules have been computed in both classical and fractional models. Our calculated vibrational energies are consistent with the Rydberg-Klein-Rees (RKR) data and previous studies. Additionally, it is seen that the vibrational energy spectra of different diatomic molecules calculated with fitted fractional parameters are improved compared to those obtained in the classical case for modelling the observed RKR data. The analysis of absolute percentage deviations at each level indicates that, for all examined diatomic molecules, the fractional derivative framework produces smaller and more consistent vibrational energy errors compared to the classical limit as the quantum number increases. Consequently, this study provides strong evidence that the GFNU method is a reliable and accurate technique to obtain the pure vibrational energies of various diatomic molecules.

## Introduction

For a long time, quantum mechanics and molecular physics have been focused on finding exact solutions to basic wave equations like the Schrödinger, Klein-Gordon, and Dirac equations. These solutions are essential for characterizing quantum systems, particularly the vibrational spectra of diatomic molecules^1–5^. In these studies, the choice of the interaction potential is crucial, as numerous empirical and theoretical models have been formulated to describe interactions in diatomic molecules. Diatomic molecules are a crucial area of study in molecular and chemical physics, significantly influencing the understanding of molecular interactions, spectroscopic characteristics, and thermodynamic behavior. The accuracy of potential models is important for predicting vibrational and rotational energy levels. These models are also important for understanding molecular dynamics and interpreting experimental spectra. Among the many empirical potentials proposed over the years, the Morse potential (MP)^6^ has gained considerable attention due to its ability to accurately model the ro-vibrational spectra of diatomic molecules. The potential energy function for the Morse potential is expressed as^6^:

1 $$\begin{aligned} V(r)=D_e\Big (1-e^{-\alpha (r-r_e)}\Big )^2. \end{aligned}$$

where $D_e$ is the dissociation energy, $r_e$ is the equilibrium distance and ${\alpha }$ denotes the screening parameter which is given by^7^:

2 $$\begin{aligned} \alpha =\sqrt{\frac{k_e}{2D_e}}, \qquad k_e=4\mu \pi ^2 c^2 w_e^2 \end{aligned}$$

where $\mu =\frac{m_1 m_2}{m_1+m_2}$ is the reduced mass, with $m_1$ and $m_2$ being the masses of the two atoms. *c* is the speed of light, and ${w_e}$ denotes the equilibrium harmonic vibrational frequency. The solutions of wave equations with the Morse potential are exact only for the case of zero angular momentum ($J = 0$). For rotating molecules ($J \ne 0$), the centrifugal barrier makes analytical solutions impossible, thus necessitating approximate methods. Roy^8^ used the Generalized Pseudospectral (GPS) approach to find the exact rovibrational energies of diatomic molecules such as H$_2$, LiH, HCl, and CO. The GPS approach uses non-uniform spatial discretization, which makes it possible to get very accurate results even for very excited states. Zúñiga et al.^9^ created an analytical perturbation method using the Pekeris approximation. They constructed a closed-form energy expression for the H$_2$ molecule by expanding the centrifugal term around an optimum internuclear distance and employing hypervirial perturbation theory, achieving remarkable concordance with precise numerical data. Okorie and Rampho^10^ examined the Modified Shifted Morse Potential (MSMP) via the asymptotic iteration method (AIM) to derive eigensolutions. Their results for the sodium dimer (Na$_2$) showed good agreement with experimental RKR data. The Dirac equation with scalar and vector potentials has also been solved for the Morse potential under conditions of pseudospin and spin symmetries.

Berkdemir^11^ investigated the Dirac equation with the Morse potential under exact pseudospin symmetry. Using the Nikiforov–Uvarov (NU) method and the Pekeris approximation for the spin–orbit coupling term, analytical bound-state solutions were obtained. In the same vein, Njoku^12^ employed the Formula Method to solve the Dirac equation for the Morse potential in the spin symmetry limit. He calculated the vibrational and rotational energies of numerous diatomic molecules, such as HI$_2$, LiH, HCl, CO, ScH, ScN, ScF, and I$_2$. The rotating Morse potential for arbitrary l-states was solved by Bayrak and Boztosun^13^ using the AIM approach. They derived energy eigenvalues and eigenfunctions for diatomic molecules, including H$_2$, HCl, CO, and LiH. Their results were in good agreement with those obtained from supersymmetry, hypervirial perturbation, and NU methods. The NU method was also employed by Berkdemir and Han^14^ to obtain bound-state solutions for the rotating Morse potential. The results for CO and LiH were in close agreement with those obtained from the variational and 1/*N* expansion methods. Soylu et al.^15^ expanded the investigation of the Morse potential by integrating isotropic velocity-dependent potentials. They obtained analytical energy spectra and demonstrated that the velocity-dependent terms substantially influence the eigenvalues, with imaginary eigenvalues indicating resonance states under certain conditions, using the AIM method.

The Gordon numerical approach was employed by Selg and Belous^16^ to solve the Schrödinger equation (SE) under a Morse-type reference potential. This approach resulted in a higher degree of accuracy and larger integration steps than the Numerov scheme. Shui and Jia^17^ solved the Dirac equation with the Morse potential to explore relativistic effects on rotational-vibrational energies. Using a Pekeris-type approximation for the centrifugal term and supersymmetric quantum mechanics, they derived a relativistic energy equation. Mirzanejad and Varganov^7^ provided a theoretical derivation of the Morse potential from an atomic screened-charge model, expressing the bond dissociation energy as a combination of electrostatic and covalent interactions. The eigenenergies for the rotating Morse potential were derived using the AIM approach by Al-Dossary^18^. The results were found to be in good agreement with those of other methods, such as supersymmetric quantum mechanics and the NU method. Numerical techniques have been increasingly employed to solve the Schrödinger equation for Morse-type potentials, particularly when analytical solutions are intractable. Sharma and Sastri^19^ used a matrix method that implemented a Fourier sine basis within an infinite spherical well to compute the ro-vibrational energies of HCl. They combined this with a Variational Monte Carlo approach to optimize Morse potential parameters by minimizing the $\chi ^2$ error between simulated and experimental vibrational frequencies. The reduction of mean percentage errors was indicative of a significant improvement in comparison to conventional multiple regression model fits, as indicated by their findings. Sastri et al.^20^ demonstrated that the time-independent Schrödinger equation for the Morse potential was solved using matrix algorithms in a Gnumeric worksheet. By deriving relationships between Morse parameters and spectroscopic constants from NIST data, they obtained vibrational frequency accuracies of within 0.02$\%$ for molecules such as HF, HBr, HI, CO, and NO. Recent theoretical studies^21–23^ have used various techniques to accurately determine the vibrational energy levels and dissociation energies of diatomic molecules, such as quantum mechanical perturbation theory and semi-classical approaches to solve the Schrödinger equation via the Morse and anharmonic potentials. Furthermore, numerous other investigations have focused on bound-state solutions in both relativistic and non-relativistic frameworks under the Morse potential^24–31^.

Recent years have seen the emergence of fractional calculus as a powerful mathematical instrument for the generalization of classical differential equations to non-integer orders. This has provided new perspectives for the prediction of the energy eigenvalues of diatomic molecules. A diverse array of fractional derivative definitions, such as those of Riemann-Liouville, Caputo, and conformable fractional derivatives, have been employed to address quantum mechanical issues. Abu-Shady and Kaabar^32^ have recently introduced a generalized fractional derivative (GFD) that maintains fundamental properties, including the product and chain principles, thereby offering a more adaptable framework for fractional quantum mechanics. Abu-Shady et al.^2^ employed the generalized fractional Nikiforov-Uvarov (GFNU) method and the GFD method to solve the *N*-dimensional radial Schrödinger equation with the Deng–Fan potential. The fractional parameter significantly impacted the rovibrational energy spectra of a number of diatomic molecules, as demonstrated by the analytical expressions they derived for the energy eigenvalues and wave functions. The study’s findings suggested that energy levels increase as both the fractional parameter and the spatial dimension *N* increase. Furthermore, the fractional model produces a more constrained energy profile than the classical case. Abu-Shady and Khokha^3^ further extended the GFD approach to the enhanced Tietz potential by solving the *D*-dimensional Schrödinger equation using the GFNU method. They computed vibrational energy levels for a wide range of diatomic molecules and proved that the GFD approach provides a more accurate representation of experimental RKR data than the classical model. Motivated by the ability of the GFD method to improve the rovibrational spectroscopy of diatomic molecules, we will devote attention to the energy spectra of diatomic molecules under the Morse Potential within the framework of the nonrelativistic wave equation. The remaining parts of this article are organized as follows. In “The fundamentals of the GFNU method”, we will introduce the GFD technique, and its application to the Schrödinger equation under the molecular Morse potential will be presented in “Bound state solution for the Morse potential in ${D}$ dimensions”. The discussion is presented in “Discussion”, and we give the conclusion in “Conclusion”.

## The fundamentals of the GFNU method

This section provides an introduction to the fundamentals of the GFNU method for the solution of the generalized fractional differential equation, which is expressed in the following form^2,3^:

3 $$\begin{aligned} D^\rho [D^\rho G(u)]+\frac{\hat{\lambda }(u)}{y(u)}D^\rho G (u)+\frac{\tilde{y}(u)}{y^2(u)}G (u)=0, \end{aligned}$$

where ${\tilde{y}(u)}$ and *y*(*u*) are polynomials of maximum ${2\rho }$-th degree and ${\lambda (u)}$ is a function at most ${\rho }$-th degree. Employing the fundamental principles of the GFD^32^

4 $$\begin{aligned} D^\rho G(u)=Hu^{1-\rho }G'(u), \end{aligned}$$

5 $$\begin{aligned} D^\rho [D^\rho G(u)]=H^2\Big [u^{2(1-\rho )}G''(u)+(1-\rho )u^{1-2\rho }G'(u)\Big ], \end{aligned}$$

where

6 $$\begin{aligned} H=\frac{\Gamma (\sigma )}{\Gamma (\sigma -\rho +1)}, \end{aligned}$$

with

7 $$\begin{aligned} 0<\rho \leqslant 1, \qquad \sigma \in R^+. \end{aligned}$$

Substituting Eqs. (4) and (5) into Eq. (3) yields

8 $$\begin{aligned} G''(u)+\frac{H(1-\rho )u^{-\rho }y(u)+\hat{\lambda }(u)}{Hu^{1-\rho }y(u)}G' (u)+\frac{\tilde{y}(u)}{H^2u^{2-2\rho }y^2(u)}G(u)=0, \end{aligned}$$

Eq. (3) can be converted to the hypergeometric equation illustrated below:

9 $$\begin{aligned} G''(u)+\frac{\hat{\lambda }_{GF}(u)}{y_{GF}(u)}G' (u)+\frac{\tilde{y}(u)}{y_{GF}^2(u)}G(u)=0, \end{aligned}$$

where

10 $$\begin{aligned} \hat{\lambda }_{GF}(u)=H(1-\rho )u^{-\rho }y(u)+\hat{\lambda }(u), \qquad y_{GF}(u)=Hu^{1-\rho }y(u). \end{aligned}$$

where the subscript GF represents the generalized fractional. Now using

11 $$\begin{aligned} G (u)= R(u) Z(u). \end{aligned}$$

Combining equations (11) and (9) yields

12 $$\begin{aligned} y_{GF}(u) Z''(u)+\lambda _{GF}(u) Z'(u)+g(u) Z(u)=0. \end{aligned}$$

where *R*(*u*) is defined as:

13 $$\begin{aligned} R(u)=\text {exp}\Big (\int \frac{\omega _{GF} (u)}{y_{GF} (u)} \, du\Big ). \end{aligned}$$

and

14 $$\begin{aligned} g(u)=\eta (u)+\omega _{GF}'(u). \end{aligned}$$

The function ${Z(u)}=Z_n (u)$ is a hypergeometric function characterized by polynomial solutions derived from the Rodrigues formula.

15 $$\begin{aligned} Z_n (u)=\frac{C_n}{\theta (u)}\frac{d^n}{du^n}[y_{GF}^n (u) \theta (u)], \end{aligned}$$

where ${C_n}$ denotes the normalization constant, and ${\theta (u)}$ represents the weight function defined as:

16 $$\begin{aligned} \theta (u)=\Big [y_{GF}(u)\Big ]^{-1}\text {exp}\Big (\int \frac{\lambda _{GF} (u)}{y_{GF} (u)} \, du\Big ). \end{aligned}$$

The polynomial ${\omega _{GF}(u)}$ is defined as:

17 $$\begin{aligned} \omega _{GF}(u)=\frac{y_{GF}'(u)-\hat{\lambda }_{GF}(u)}{2}\pm \sqrt{\Bigg [\frac{y_{GF}'(u)-\hat{\lambda }_{GF}(u)}{2}\Bigg ]^2-\tilde{y}(u)+\eta (u)y_{GF}(u)}, \end{aligned}$$

The function ${\eta (u)}$ can be derived if the expression within the square root is the square of a polynomial. Therefore, the eigenvalue formula is:

18 $$\begin{aligned} \xi (u)=\xi _n(u)=-n\Big [\lambda _{GF}'(u)+\frac{(n-1)}{2}y_{GF}''(u)\Big ], \end{aligned}$$

where

19 $$\begin{aligned} \lambda _{GF}(u)=\hat{\lambda }_{GF}(u)+2\omega _{GF}(u). \end{aligned}$$

Subsequently, the eigenfunctions *G*(*u*) can be obtained by inserting Eqs. (13) and (15) into Eq. (11).

## Bound state solution for the Morse potential in *D* dimensions

The *D*-dimensional radial SE for a DM with the potential *V*(*r*) is provided by^1,2^.

20 $$\begin{aligned} \psi ''(r)+\frac{D-1}{r}\psi '(r) +\Biggl \{\frac{2\mu }{\hbar ^2}\Big (E-V(r)\Big )-\frac{J(J+D-2)}{r^2}\Biggl \}\psi (r)=0, \end{aligned}$$

where *E* represent the energy eigenvalue, *D* is the number of dimensions, and *J* is the vibrational quantum number, respectively, while ${\hbar }$ denotes the reduced Planck’s constant. By using,

21 $$\begin{aligned} \psi (r)=r^\frac{1-D}{2} \phi (r). \end{aligned}$$

Eq. (20) becomes

22 $$\begin{aligned} \phi ''(r)+\Bigg [\frac{2\mu }{\hbar ^2}\Big (E-V(r)\Big )-\frac{(\gamma ^2-\frac{1}{4})}{r^2}\Bigg ]\phi (r)=0, \end{aligned}$$

with

23 $$\begin{aligned} \gamma =J+\frac{D-2}{2}. \end{aligned}$$

By adding the Morse potential (1) into Eq. (22) yields:

24 $$\begin{aligned} \phi ''(r)+\Biggl \{\frac{2\mu }{\hbar ^2}\Bigg [E-D_e\Big (1-e^{-\alpha (r-r_e)}\Big )^2-\frac{(\gamma ^2-\frac{1}{4})}{r^2}\Bigg )\Biggl \}\phi (r)=0, \end{aligned}$$

Now, by applying the Pekeris approximation^9,11,14,18^ to the centrifugal term ${(\gamma ^2-\frac{1}{4})\big /r^2}$ yields the approximate analytical solutions of Eq. (24)

25 $$\begin{aligned} \frac{\gamma ^2-\frac{1}{4}}{r^2}\approx \frac{\gamma ^2-\frac{1}{4}}{r_e^2}\Bigg [p_0+p_1e^{-\alpha r}+p_2e^{-2\alpha r}\Bigg ]. \end{aligned}$$

where the coefficients ${p_0, p_1}$ and ${p_2}$ are given below^9,11,14,18^:

26 $$\begin{aligned} p_0=1-\frac{3}{\alpha r_e}+\frac{3}{\alpha ^2 r_e^2}, \end{aligned}$$

27 $$\begin{aligned} p_1=2e^{\alpha r_e}\Bigg (\frac{2}{\alpha r_e}-\frac{3}{\alpha ^2 r_e^2}\Bigg ), \end{aligned}$$

28 $$\begin{aligned} p_2=e^{2\alpha r_e}\Bigg (\frac{3}{\alpha ^2 r_e^2}-\frac{1}{\alpha r_e}\Bigg ). \end{aligned}$$

Substituting Eq. (25) into Eq. (24) produces

29 $$\begin{aligned} \phi ''(r)+\Biggl \{\frac{2\mu }{\hbar ^2}\Bigg [E-D_e\Big (1-e^{-\alpha (r-r_e)}\Big )^2-\frac{\gamma ^2-\frac{1}{4}}{r_e^2}\Bigg [p_0+p_1e^{-\alpha r}+p_2e^{-2\alpha r}\Bigg ]\Biggl \}\phi (r)=0, \end{aligned}$$

By utilizing the transformation ${u=e^{-\alpha r}}$, Eq. (29) becomes

30 $$\begin{aligned} \phi ''(u)+\frac{(1-u)}{u(1-u)}\phi '(u)+\frac{1}{u^2(1- u)^2}\Big [-F_1u^2+F_2u-F_3\Big ]\phi (u)=0, \end{aligned}$$

where

31 $$\begin{aligned} F_1=\hat{\gamma }\big (p_0+p_1+p_2\big )+\hat{\mu } e^{-2\alpha r_e}-\hat{E}, \end{aligned}$$

32 $$\begin{aligned} F_2=\hat{\gamma }\big (2p_0+p_1\big )-2\hat{\mu } e^{-\alpha r_e}-2\hat{E}, \end{aligned}$$

33 $$\begin{aligned} F_3=\hat{\gamma } p_0+\hat{\mu }-\hat{E}, \end{aligned}$$

with

34 $$\begin{aligned} \hat{\gamma }=\frac{\gamma ^2-\frac{1}{4}}{\alpha ^2r_e^2}, \qquad \hat{\mu }=\frac{2\mu D_e}{\alpha ^2\hbar ^2}, \qquad \hat{E}=\frac{2\mu E}{\alpha ^2\hbar ^2}. \end{aligned}$$

The generalized fractional form of the SE for the Morse potential can be obtained by converting the integer orders in Eq. (30) into fractional orders.

35 $$\begin{aligned} D^\rho \big [D^\rho \phi (u)\big ]+\frac{(1- u^\rho )}{u^\rho (1- u^\rho )}D^\rho \big [\phi (u)\big ]+\frac{1}{u^{2\rho }(1- u^\rho )^2}\Big [-F_1u^{2\rho }+F_2u^\rho -F_3\Big ]\phi (u)=0, \end{aligned}$$

Substituting Eqs. (4) and (5) into Eq. (35) produces

36 $$\begin{aligned} \phi ''(u)+\frac{\Big [H(1-\rho )+1\Big ](1- u^\rho )}{Hu(1- u^\rho )}\phi '(u)+\frac{1}{H^2u^2(1- u^\rho )^2}\Big [-F_1u^{2\rho }+F_2u^\rho -F_3\Big ]\phi (u)=0, \end{aligned}$$

The following functions are obtained by comparing Eq. (36) with Eq. (9):

37 $$\begin{aligned} \tilde{\lambda }_{GF}(u)=\Big (H(1-\rho )+1\Big )(1-u^\rho ), \qquad y_{GF}(u)=Hu(1-u^\rho ), \qquad \tilde{y}_{GF}(u)=-F_1u^{2\rho }+F_2u^\rho -F_3. \end{aligned}$$

By incorporating Eq. (37) into Eq. (17), the function ${\pi _{GF}(u)}$ is determined as the following:

38 $$\begin{aligned} {\begin{matrix} & \omega _{GF}(u)=\frac{(H\rho -1)+(1-2H\rho )u^\rho }{2}\pm \\ & \sqrt{\Big [\frac{(1-2H\rho )^2}{4}+F_1- H\eta u^{1-\rho }\Big ]u^{2\rho }+\Big [\frac{(H\rho -1)(1-2H\rho )}{2}-F_2+H\eta u^{1-\rho }\Big ]u^\rho +\Big [\frac{(H\rho -1)^2}{4}+F_3\Big ]}. \end{matrix}} \end{aligned}$$

Equation (38) can be simplified to the as follows:

39 $$\begin{aligned} \omega _{GF}(u)=\frac{(H\rho -1)+(1-2H\rho )u^\rho }{2}\pm \sqrt{R_1 u^{2\rho }+R_2u^\rho +R_3}, \end{aligned}$$

where

40 $$\begin{aligned} R_1=D_1-H\eta u^{1-\rho }, \qquad R_2=D_2+H\eta u^{1-\rho }, \qquad R_3=D_3, \end{aligned}$$

with

41 $$\begin{aligned} D_1=\frac{(1-2H\rho )^2}{4}+F_1, \qquad D_2=\frac{(H\rho -1)(1-2H\rho )}{2}-F_2, \qquad D_3=\frac{(H\rho -1)^2}{4}+F_3. \end{aligned}$$

The function ${\eta (u)}$ can be derived by applying the condition that the discriminant of the function within the square root of Eq. (39) equals zero.

42 $$\begin{aligned} \eta _\pm (u)=\hat{H}\Biggl [-\big (D_2+2 D_3\big )\pm 2\sqrt{D_3\Big (D_1+D_2+D_3\Big )}\Biggl ]u^{\rho -1}; \qquad \hat{H}=\frac{1}{H}. \end{aligned}$$

Putting Eq. (42) into Eq. (39) gives

43 $$\begin{aligned} \omega _{GF}(u)=\frac{(H\rho -1)+(1-2H\rho )u^\rho }{2}\pm {\left\{ \begin{array}{ll} \Big (\sqrt{D_3}-\sqrt{D_1+D_2+D_3}\Big )u^\rho -\sqrt{D_3}, \qquad \eta =\eta _+ \\ \Big (\sqrt{D_3}+\sqrt{D_1+D_2+D_3}\Big )u^\rho -\sqrt{D_3}, \qquad \eta =\eta _- \\ \end{array}\right. }. \end{aligned}$$

In order to identify a solution that is physically feasible, we employ the negative sign in Eq. (43), which alters the value of ${\omega _{GF}(u)}$ to

44 $$\begin{aligned} \omega _{GF}(u)=\frac{(H\rho -1)+(1-2H\rho )u^\rho }{2}-\Big (\sqrt{D_3}-\sqrt{D_1+D_2+D_3}\Big )u^\rho +\sqrt{D_3}, \end{aligned}$$

and

45 $$\begin{aligned} \eta (u)=\hat{H}\Biggl [-\big (D_2+2D_3\big )+2\sqrt{D_3\Big (D_1+D_2+D_3\Big )}\Biggl ]u^{\rho -1}. \end{aligned}$$

Consequently, the functions ${\xi (u), \lambda _{GF}(u)}$ and ${\xi _n(u)}$ are expressed as follows:

46 $$\begin{aligned} \xi (u)=\Bigg [-\hat{H}\big (D_2+2D_3\big )-\sqrt{D_3}\Big ( \rho -2\hat{H}\sqrt{D_1+D_2+D_3}\Big )+\rho \Bigg (\frac{1}{2}\Big (1-2H\rho \Big )+\sqrt{D_1+D_2+D_3}\Bigg )\Bigg ]u^{\rho -1}, \end{aligned}$$

47 $$\begin{aligned} \lambda _{GF}(u)=\Big (2\sqrt{D_3}+H\Big )-\Big [H(\rho +1)+2\Big (\sqrt{D_3}-\sqrt{D_1+D_2+D_3}\Big )\Big ]u^\rho , \end{aligned}$$

48 $$\begin{aligned} \xi _n(u)=n\rho \Big [\frac{H(n+1)(\rho +1)}{2}+2\Big (\sqrt{D_3}-\sqrt{D_1+D_2+D_3}\Big )\Big ]u^{\rho -1}. \end{aligned}$$

The energy spectra of a DM in can be expressed in the fractional form by combining Eqs. (46) and (48) as:

49 $$\begin{aligned} {\begin{matrix} E_{F}=& \frac{\alpha ^2\hbar ^2}{2\mu }\Bigg [K_3+\frac{(H\rho -1)^2}{4}\Bigg ]\\ & -\frac{\alpha ^2\hbar ^2}{2\mu }\Bigg [\frac{\rho \Big (\zeta -(2n+1)\sqrt{\frac{H^2\rho ^2}{4}+K_3- K_2+K_1}\Big )+\hat{H}\Big (\frac{H\rho (1-H\rho )}{2}+2 K_3-K_2\Big )}{2\hat{H}\sqrt{\frac{H^2\rho ^2}{4}+K_3- K_2+K_1}-\rho (2n+1)}\Bigg ]^2, \end{matrix}} \end{aligned}$$

where

50 $$\begin{aligned} \zeta =\frac{1}{2}\Big [Hn(n+1)(\rho +1)+2H\rho -1\Big ], \qquad K_1=\rho \big (D_2+D_1+D_0\big )+\hat{\mu } e^{-2\alpha r_e}, \end{aligned}$$

51 $$\begin{aligned} K_2=\hat{\gamma }\big (D_1+2D_0\big )+2\hat{\mu } e^{-\alpha r_e}, \qquad K_3=\hat{\gamma } D_0+\hat{\mu }. \end{aligned}$$

By setting ${\rho =\sigma =1}$ yields the following classical equation for the energy spectra in the lack of fractional parameters:

52 $$\begin{aligned} E_{C}=\frac{\alpha ^2\hbar ^2}{2\mu }\Biggl \{D_3-\frac{\big [n(n+1)+\frac{1}{2}\big ]-(2n+1)\sqrt{D_3- D_2+D_1+\frac{1}{4}}+2 D_3-D_2}{\sqrt{D_3-D_2+D_1+\frac{1}{4}}-(2n+1)}\Biggl \}^2. \end{aligned}$$

By employing Eq. (13), the function *R*(*u*) is transformed into

53 $$\begin{aligned} R(u)=u^{\hat{H}\Big (\frac{(H\rho -1)}{2}+\sqrt{D_3}\Big )}\Big (1- u^\rho \Big )^{\Big ({\frac{1}{2}-\frac{\hat{H}}{\rho }\sqrt{D_1+D_2+D_3}}\Big )}. \end{aligned}$$

Utilizing Eq. (16), the function ${\rho (u)}$ can be expressed below:

54 $$\begin{aligned} \theta (u)=\hat{H} u^{2\hat{H}\sqrt{D_3}}\Big (1- u^\rho \Big )^{{-\frac{2\hat{H}}{\rho }\sqrt{D_1+D_2+D_3}}}. \end{aligned}$$

Using Eq. (15), we can write the function ${Z_n(u)}$ as:

55 $$\begin{aligned} Z_n (u) =C_n u^{-2\hat{H}\sqrt{D_3}}\Big (1- u^\rho \Big )^{{\frac{2\hat{H}}{ \rho }\sqrt{D_1+D_2+D_3}}}\frac{d^n}{du^n}\Bigg [H^n u^{\Big (n+2\hat{H}\sqrt{D_3}\Big )}\Big (1- u^\rho \Big )^{{\Big (n-\frac{2\hat{H}}{ \rho }\sqrt{D_1+D_2+D_3}\Big )}}\Bigg ]. \end{aligned}$$

The solution of Eq. (30) is derived by utilizing Eq. (11) as demonstrated below:

56 $$\begin{aligned} \phi (u)=C_n u^{\hat{H}\Big (\frac{(H\rho -1)}{2}-\sqrt{D_3}\Big )}\Big (1-u^\rho \Big )^{\Big ({\frac{1}{2}+\frac{\hat{H}}{ \rho }\sqrt{D_1+D_2+D_3}}\Big )}\frac{d^n}{du^n}\Bigg [H^n u^{\Big (n+2\hat{H}\sqrt{D_3}\Big )}\Big (1- u^\rho \Big )^{{\Big (n-\frac{2\hat{H}}{ \rho }\sqrt{D_1+D_2+D_3}\Big )}}\Bigg ]. \end{aligned}$$

**Figure 1:**
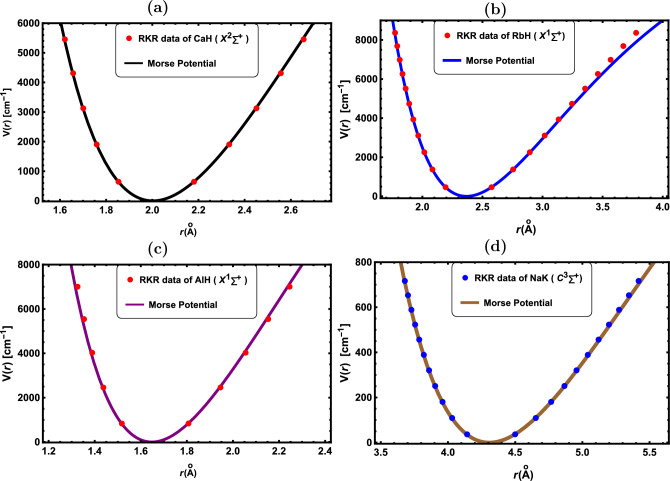
RKR data points and Morse potential for the: (**a**) CaH(${X^2\Sigma ^+}$), (**b**) RbH(${X^1\Sigma ^+}$), (**c**) AlH(${X^1\Sigma ^+}$) and (**d**) NaK(${c^3\Sigma ^+}$).

**Figure 2:**
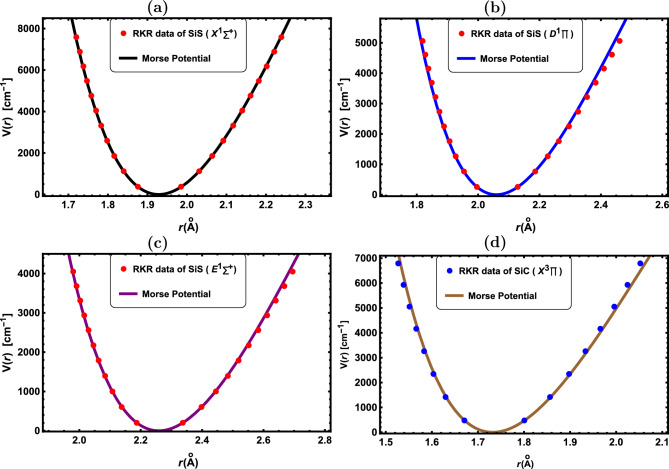
RKR data points and Morse potential for the: (**a**) SiS(${X^1\Sigma ^+}$), (**b**) SiS(${D^1\Pi }$), (**c**) SiS(${E^1\Sigma ^+}$) and (**d**) SiC(${X^3\Pi }$).

**Figure 3:**
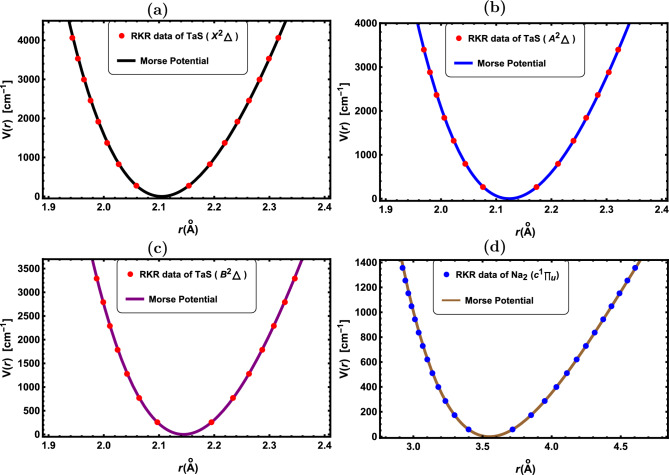
RKR data points and Morse potential for the: (**a**) TaS(${X^2\Delta }$), (**b**) TaS(${A^2\Delta }$), (**c**) TaS(${B^2\Delta }$) and (**d**) Na$_2$(${c^1\Pi _u}$).

**Figure 4:**
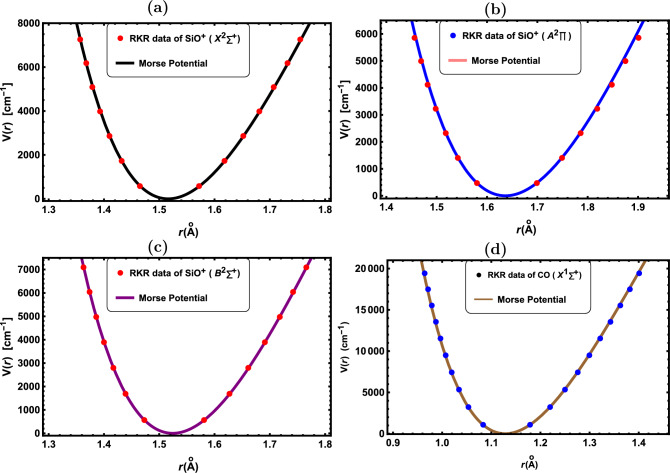
RKR data points and Morse potential for the: (**a**) SiO${^+}$(${X^2\Sigma ^+}$), (**b**) SiO${^+}$(${A^2\Pi }$), (**c**) SiO${^+}$(${B^2\Sigma ^+}$) and (**d**) CO(${X^1\Sigma ^+}$).

**Figure 5:**
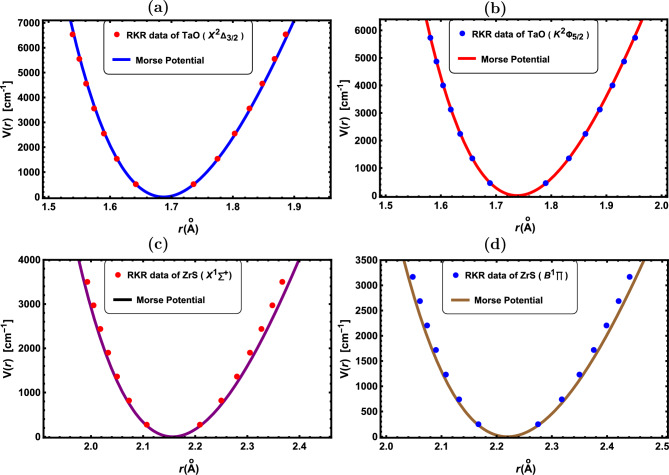
RKR data points and Morse potential for the: (**a**) TaO(${X^2\Delta }$), (**b**) TaO(${K^2\Phi }$), (**c**) ZrS(${X^1\Sigma ^+}$) and (**d**) ZrS(${B^1\Pi }$).

## Discussion

The results derived in the previous section are applied to a variety of diatomic molecules, including CaH $(X^2\Sigma ^+)$, RbH $(X^1\Sigma ^+)$, AlH $(X^1\Sigma ^+)$, SiC $(X^3\Pi )$, NaK $(c^3\Sigma ^+)$, Na$_2$ $(c^1\Pi _u)$, CO $(X^1\Sigma ^+)$, ZrS $(X^1\Sigma ^+)$, ZrS $(B^1\Pi )$, TaO $(X^2\Delta _{3/2})$, TaO $(K^2\Phi _{5/2})$, SiS $(X^1\Sigma ^+)$, SiS $(D^1\Pi )$, SiS $(E^1\Sigma ^+)$, TaS $(X^2\Delta )$, TaS $(A^2\Delta )$, TaS $(B^2\Delta )$, SiO $^+$ $(X^2\Sigma ^+)$, SiO $^+$ $(A^2\Pi )$, SiO $^+$ $(B^2\Sigma ^+)$, CS $(X^1\Sigma ^+)$ and CN $(X^2\Sigma ^+)$. We have chosen these molecules for their importance in the fields of quantum chemistry, material science, and molecular physics. Initially, the Morse potential is employed to generate the potential function curves for the considered diatomic molecules. Table 1 displays the molecular parameters that were employed in this investigation, which were obtained from the literature^35–42^ in addition to the fractional parameters ($\rho$ and $\sigma$).

**Table 1:** Molecular constants^35–42^ and fraction parameters for the selected diatomic molecules.

Molecule	Molecular constants			Fraction parameters	
	${\omega _e}$ ${(cm^{-1})}$	${r_e}$ (${\overset{o}{A}}$)	${D_e}$(eV)	$\rho$	$\sigma$
$\text {CaH}\, (X^2\Sigma ^+)$	1298.34	2.0025	2.80	0.8642	0.6908
$\text {RbH}\, (X^1\Sigma ^+)$	937.10	2.3668	1.81	0.8293	0.6428
$\text {AlH}\, (X^1\Sigma ^+)$	1682.56	1.6478	3.10	0.8902	0.7075
$\text {SiC}\, (X^3\Pi )$	954.20	1.7320	3.78	0.8093	0.6420
$\text {NaK}\, (c^3\Sigma ^+)$	73.40	4.3075	0.31	0.8956	0.6664
$\text {Na}_2 \,(c^1\Pi _u)$	116.31	3.5503	0.69	0.8187	0.6594
$\text {CO}\, (X^1\Sigma ^+)$	2169.82	1.1283	11.24	0.8254	0.6612
$\text {ZrS}\, (X^1\Sigma ^+)$	548.34	2.1566	5.89	0.7856	0.6303
$\text {ZrS}\, (B^2\Pi )$	495.92	2.2195	5.89	0.7866	0.6327
$\text {TaO}\, (X^2\Delta _{3/2})$	1028.90	1.6873	8.19	0.7907	0.6338
$\text {TaO}\, (K^2\Phi _{5/2})$	905.45	1.7382	8.19	0.8253	0.6699
$\text {SiS}\, (X^1\Sigma ^+)$	749.64	1.9286	6.76	0.8648	0.6873
$\text {SiS}\, (D^1\Pi )$	512.66	2.0596	3.19	0.7726	0.6290
$\text {SiS}\, (E^1\Sigma ^+)$	405.60	2.2589	3.41	0.9393	0.8675
$\text {TaS}\, (X^2\Delta )$	551.98	2.1049	6.90	0.8906	0.7088
$\text {TaS}\, (A^2\Delta )$	531.27	2.1234	6.90	0.7580	0.6131
$\text {TaS}\, (B^2\Delta )$	514.80	2.1444	6.90	0.7725	0.6246
$\text {SiO}^+\,(X^2\Sigma ^+)$	1162.18	1.5162	5.75	0.7877	0.6313
$\text {SiO}^+\,(A^2\Pi )$	946.28	1.6365	5.75	0.7640	0.6301
$\text {SiO}^+\, (B^2\Sigma ^+)$	1136.58	1.5243	5.75	0.7877	0.6332
$\text {CS}\,(X^1\Sigma ^+)$	1285.08	1.5349	7.35	0.8419	0.6686
$\text {CN}\,(X^2\Sigma ^+)$	2068.59	1.1718	7.81	0.9499	0.6951

**Table 2:** Vibrational energies (${cm^{-1}}$) for NaK (${c^3\Sigma ^+}$) molecule.

*n*	RKR^35^	Ref.^44^	Eq. (52)	Eq. (49)
0	36.59	36.57	36.57	37.01
1	109.02	108.94	108.89	110.19
2	180.49	180.26	180.15	182.28
3	250.98	250.54	250.32	253.27
4	320.49	319.77	319.43	323.15
5	389.02	387.95	387.46	391.94
6	456.55	455.08	454.41	459.62
7	523.08	521.17	520.30	526.21
8	588.61	586.20	585.10	591.69
9	653.13	650.17	648.84	656.08
10	716.63	713.09	711.50	719.36
11	779.11	774.96	773.08	781.55
12	840.56	835.76	833.60	842.63
13	900.98	895.51	893.03	902.62
14	960.35	954.20	951.40	961.50
15	1018.69	1011.82	1008.69	1019.29
16	1075.97		1064.90	1075.97
17	1132.19		1120.04	1131.55
18	1187.35		1174.11	1186.04
19	1241.44		1227.10	1239.42
20	1294.45		1279.02	1291.70
21	1346.38		1329.86	1342.89
22	1397.23		1379.64	1392.97
23	1446.98		1428.33	1441.95
24	1495.63		1475.95	1489.83
25	1543.18		1522.50	1536.62
26	1589.62		1567.98	1582.30
27	1634.94		1612.38	1626.88
28	1679.14		1655.70	1670.36
29	1722.21		1697.96	1712.74
30	1764.15		1739.13	1754.02
31	1804.95		1779.24	1794.21
32	1844.60		1818.27	1833.29
33	1883.09		1856.22	1871.27
34	1920.44		1893.10	1908.15
35	1956.61		1928.91	1943.93
36	1991.62		1963.64	1978.61
MAPD%			0.9788	0.4834

In Figs. 1, 2, 3, 4 and 5, the experimental RKR points for the selected diatomic molecules have been shown together with the potential function curves produced by the Morse potential. In all instances, the Morse potential curve closely aligns with the experimental potential energy curve, revealing that the Morse potential model yields a dependable interpretation of molecular interactions.

In order to verify the analytical expressions obtained for the Morse potential using the GFNU technique, the pure vibrational energy spectra were calculated for a selection of diatomic molecules. The computed energy eigenvalues are displayed in Tables 2, 3, 4, 5, 6, 7, 8, 9, 10, 11, and 12, in conjunction with experimental RKR data and findings from previous investigations for comparative analysis. To enhance the assessment of the correctness and precision of the current model, the mean absolute percentage deviation (MAPD) of the Morse potential energies from the experimental RKR data points was calculated, as specified below^45^:

57 $$\begin{aligned} \text {MAPD}=\frac{100}{N}\sum _n \Bigg |1-\frac{E_{nJ}}{E_{RKR}}\Bigg |, \end{aligned}$$

where ${E_{RKR}}$ denotes the experimental RKR data point, and ${E_{nJ}}$ are the corresponding vibrational energy eigenvalues determined by the Morse potential model for a given vibrational quantum number (*n*) and rotational quantum number (*J*).

**Table 3:** Vibrational energies (${cm^{-1}}$) for Na$_2$ (${c^1\Pi _u}$) molecule.

*n*	RKR^36^	SPTP^45^	IPTP^45^	Eq. (52)	Eq. (49)
0	58.005	58.010	58.008	58.236	58.128
1	173.023	173.106	173.104	173.331	173.012
2	286.764	286.988	286.986	287.213	286.687
3	399.242	399.657	399.655	399.881	399.152
4	510.472	511.112	511.110	511.336	510.408
5	620.468	621.354	621.352	621.576	620.455
6	729.244	730.381	730.379	730.603	729.293
7	836.815	838.195	838.193	838.416	836.921
8	943.196	944.796	944.794	945.016	943.341
9	1048.401	1050.182	1050.180	1050.402	1048.551
10	1152.445	1154.355	1154.353	1154.574	1152.551
11	1255.343	1257.314	1257.312	1257.532	1255.343
12	1357.106	1359.059	1359.058	1359.277	1356.925
MAPD%		0.1257	0.1251	0.1939	0.0272

**Table 4:** Vibrational energies (${cm^{-1}}$) for RbH (${X^1\Sigma ^+}$) molecule.

*n*	RKR^37^	NU^48^	WKB^48^	Eq. (52)	Eq. (49)
0	465.07	464.727	468.429	464.79	468.98
1	1373.86	1371.75	1375.32	1371.78	1383.95
2	2254.98	2248.65	2252.09	2248.66	2268.26
3	3108.91	3095.43	3098.75	3095.42	3121.89
4	3936.14	3912.1	3915.29	3912.07	3944.86
5	4737.17	4698.65	4701.71	4698.6	4737.17
6	5512.47	5455.08	5458.02	5455.02	5498.81
7	6262.54	6181.4	6184.21	6181.32	6229.78
8	6987.87	6877.6	6880.28	6877.50	6930.08
9	7688.94	7543.68	7546.23	7543.57	7599.72
10	8366.25	8179.64	8182.07	8179.52	8238.70
MAPD%		0.9455	0.9452	0.9448	0.6443

**Table 5:** Vibrational energies (${cm^{-1}}$) for SiC (${X^3\Pi }$) molecule.

*n*	RKR^38^	NU^28^	Eq. (52)	Eq. (49)
0	475.47	475.02	476.57	478.14
1	1416.67	1412.57	1415.83	1420.49
2	2344.87	2333.46	2340.16	2347.80
3	3260.07	3237.70	3249.56	3260.08
4	4162.27	4125.29	4144.03	4157.32
5	5051.47	4996.22	5023.56	5039.54
6	5927.67	5850.50	5888.16	5906.72
7	6790.67	6688.12	6737.83	6758.87
MAPD%		0.7942	0.4064	0.2671

The best-fit values of the fractional parameters ($\rho$ and $\sigma$) are determined by using the *FindMinimum* function in the *MATHEMATICA* software to calculate the minimization of the MAPD in Eq. (57) based on the available RKR data points for each molecular state. In a complicated many-body system, an effective, non-local connection between the nuclei of a diatomic molecule can be produced via interactions with electrons and surrounding degrees of freedom. Consequently, the fractional order ($0<\rho \leqslant 1$) serves not only as a fitting parameter but also as a measurable indicator of the non-locality or anomalous character of the quantum system. The parameter $\sigma$, derived from the GFD specification (6), serves as a scaling and calibrating factor to ensure the dimensional and mathematical consistency of the fractional derivative operator.

In both the classical and fractional scenarios, the vibrational energies of the considered diatomic molecules are evaluated using Eqs. (52) and (49) in three-dimensional space ($D = 3$). The vibrational energies derived from the Morse potential are in good alignment with the experimental RKR data, as indicated in Tables 2, 3, 4, 5, 6, 7, 8, 9, 10, 11, and 12. Moreover, the estimated MAPD implies that there exists a ${1\%}$ error margin relative to the experimental RKR values across every specified diatomic molecule. Table 2 illustrates the vibrational energies of the NaK (${c^3\Sigma ^+}$) molecule, which are very consistent with the RKR data^35^. Our fractional model (Eq. (49)) demonstrates higher precision and yields an overall MAPD of 0.4834$\%$, compared to the classical model (Eq. (52), which has a MAPD of 0.9788$\%$. A particular comparison of previous investigation^44^ (up to *n*=15) reveals that our classical model using Eq. (52) (MAPD=0.5459$\%$) corresponds with theoretical work^44^ (MAPD=0.3750$\%$). Table 3 illustrates the vibrational energy data for the Na$_2$ (${c^1\Pi _u}$) molecule, which lets us compare how well the current model works. The results demonstrate that the fractional situation (Eq. (52)) is more accurate, with a very low MAPD of 0.0272$\%$ compared to the experimental RKR data^36^. In the classical case, our findings are in satisfactory accord with other potential models, including the Improved Pöschl–Teller potential (IPTP) and the Simplified Pöschl–Teller potential (SPTP). Moreover, the calculated vibrational energies for the RbH (${X^1\Sigma ^+}$) molecule, listed in Table 4, also indicate how accurate the current model is, with the fractional case (Eq. (49)) exhibiting the best results. The energies calculated using Eq. (49) align properly with the experimental RKR data^37^ for all vibrational quantum states. The MAPD confirms this statistically, with the fractional model getting a considerably lower result of 0.6443$\%$. Additionally, our results in the classical case are exactly the same as those obtained with the NU and WKB methods utilizing the deformed hyperbolic potential^48^. In the same way, the results for the SiC (${X^3\Pi }$) molecule in Table 5 show that the current model fits the RKR data^38^ more successfully. The fractional case (Eq. (49)) subsequently provides the most accurate results, with a MAPD of just 0.2671$\%$. Our MAPD in the classical limit (Eq. (52)), which is 0.4064$\%$, outperforms the value obtained for the shifted Morse potential using the NU approach^28^, which is 0.7942$\%$. In Table 6, we have reported our vibrational energies for the CO (${X^1\Sigma ^+}$) molecule alongside the experimental RKR data and the theoretical results from Refs.^46,47^ and^49^. In the classical case, our results are systematically compared with those of Refs.^46,47^ and^49^. over different vibrational ranges, demonstrating a consistently good level of agreement.

**Table 6:** Vibrational energies (${cm^{-1}}$) for CO (${X^1\Sigma ^+}$) molecule.

*n*	RKR^39^	MHTP^46^	IGPTP^47^	ITP^49^	IPTP^49^	Eq. (52)	Eq. (49)
0	1081.7756312	1081.9289	1081.733857	1081.654643	1081.929909	1081.67514360	1081.20040751
1	3225.0467372	3225.7758	3225.582747	3224.422665	3225.776751	3225.55284916	3224.14577993
2	5341.8378022	5343.6600	5343.468880	5340.186077	5343.660903	5343.46711253	5341.15056389
3	7432.2146800	7435.5813	7435.392256	7428.988575	7435.582261	7435.41793371	7432.21475940
4	9496.2449180	9501.5400	9501.352875	9490.867414	9501.540929	9501.40531271	9497.33836644
5	11533.997773	11541.5358	11541.350737	11525.86578	11541.53670	11541.4292495	11536.521385
6	13545.544213	13555.5689	13555.385841	13534.02295	13555.56978	13555.4897441	13549.7638151
7	15530.956905	15543.6392	15543.458188	15515.38191	15543.64007	15543.5867966	15537.0656568
8	17490.310178	17505.7467	17505.567778	17469.97981	17505.74757	17505.7204068	17498.4269100
9	19423.679979	19441.8915	19441.714610	19397.85929	19441.89237	19441.8905748	19433.8475747
10	21331.143801	21352.0735	21351.898686	21299.06118	21352.07439	21352.0973007	21343.3276510
11	23212.780596	23236.2928	23236.120004	23173.62429	23236.29361	23236.3405844	23226.8671389
12	25068.670663	25094.5493	25094.378565	25021.58728	25094.55009	25094.6204259	25084.4660382
13	26898.895524	26926.8430	26926.674368	26842.99431	26926.84378	26926.9368252	26916.1243491
14	28703.537765	28733.1739	28733.007415	28637.88207	28733.17477	28733.2897823	28721.8420716
15	30482.680865	30513.5421	30513.377703	30406.29078	30513.54293	30513.6792972	30501.6192055
16	32236.4089982	32267.9475	32267.785238	32148.26073	32267.94834	32268.1053699	32255.4557511
17	33964.8068144	33996.3902	33996.230010	33863.83192	33996.39091	33996.5680005	33983.3517081
18	35667.9591954	35698.8701	35698.712028	35553.04420	35698.87084	35699.0671888	35685.3070767
19	37345.9509881	37375.3872	37375.231288	37215.93425	37375.38790	37375.6029350	37361.3218569
20	38998.8667139	39025.9415	39025.787791	38852.54505	39025.94226	39026.1752390	39011.3960486
21	40626.7902542	40650.5331	40650.381537	40462.91454	40650.53387	40650.7841008	40635.5296518
22	42229.8045118	42249.1619	42249.012526	42047.08079	42249.16265	42249.4295204	42233.7226666
23	43807.9910479	43821.8280	43821.680757	43605.08322	43821.82868	43822.1114978	43805.9750929
24	45361.4296943		45368.386231	45136.96127	45368.53197	45368.8300330	45352.2869307
25	46890.1981405		46889.128948	46642.75409	46889.27249	46889.5851260	46872.6581801
26	48394.3714951		48383.908908	48122.49943	48384.05024	48384.3767769	48367.0888410
27	49874.0218206		49852.726110	49576.23777	49852.86517	49853.2049855	49835.5789135
28	51329.2176409		51295.580555	51004.00523	51295.71737	51296.0697520	51278.1283975
29	52760.0234202		52712.472243	52405.84349	52712.60689	52712.9710763	52694.7372930
30	54166.4990111		54103.401174	53781.78715	54103.53351	54103.9089584	54085.4056001
31	55548.6990707		55468.367347	55131.87761	55468.49745	55468.8833983	55450.1333187
32	56906.6724427		56807.370763	56456.15091	56807.49862	56807.8943960	56788.9204489
33	58240.4615006		58120.411422	57754.64792	58120.53691	58120.9419515	58101.7669906
34	59550.1014507		59407.489324	59027.40332	59407.61256	59408.0260649	59388.6729439
35	60835.6195899		60668.604469	60274.45785	60668.72544	60669.1467360	60649.6383087
36	62097.0345120		61903.756856	61495.84733	61903.87555	61904.3039650	61884.6630850
37	63334.3552600		63112.946486	62691.61096	63113.06284	63113.4977518	63093.7472728
38	64547.5804100			63861.78558	64296.28737	64296.7280964	64276.8908723
39	65736.6970930			65006.40912	65453.54915	65453.9949987	65434.0938832
40	66901.6799240			66125.51833	66584.84816	66585.2984590	66565.3563057
41	68042.4898490			67219.15194	67690.18440	67690.6384770	67670.6781397
MAPD%				0.4685	0.1336	0.1330	0.1274

**Table 7:** Vibrational energies (${cm^{-1}}$) for the (${X^2\Sigma ^+}$), (${A^2\Pi }$) and (${B^2\Sigma ^+}$) states of SiO$^+$ molecule.

*n*	${X^2\Sigma ^+}$			${A^2\Pi }$			${B^2\Sigma ^+}$		
	RKR^40^	Eq. (52)	Eq. (49)	RKR^40^	Eq. (52)	Eq. (49)	RKR^40^	Eq. (52)	Eq. (49)
0	579.3	579.3	579.8	471.3	473.2	469.1	566.4	566.6	566.5
1	1727.5	1726.9	1728.5	1403.6	1409.8	1397.5	1688.9	1689.2	1689.1
2	2861.8	2860.0	2862.7	2321.9	2336.8	2316.6	2797.6	2798.0	2797.7
3	3982.2	3978.5	3982.2	3226.1	3254.1	3226.1	3892.4	3892.8	3892.4
4	5088.6	5082.4	5087.1	4116.4	4161.8	4126.1	4973.4	4973.6	4973.2
5	6181.1	6171.8	6177.5	4992.6	5059.8	5016.7	6040.6	6040.6	6040.0
6	7259.7	7246.6	7253.3	5854.8	5948.1	5897.8	7093.9	7093.6	7093.0
MAPD%		0.0926	0.0511		0.9124	0.3704		0.0110	0.0082

For levels up to $n = 23$, our classical energies obtained from Eq. (52)) yield a MAPD of 0.0722$\%$, which is in excellent agreement with the 0.0728$\%$ MAPD reported for the modified Hyperbolical-Type potential (MHTP)^46^. Extending the range to $n = 37$, our classical model maintains this accuracy, with a MAPD of 0.0994$\%$ compared to the 0.0990$\%$ MAPD of the improved generalized Pöschl–Teller potential (IGPTP)^47^. Over the full vibrational range up to $n = 41$, our classical model achieves a MAPD of 0.1329$\%$, remaining in close agreement with the improved Pöschl–Teller potential (IPTP) (0.1336$\%$) and a good enhancement with the improved Tietz potential (ITP) (0.4685$\%$) reported in Ref.^49^.

Tables 7 and 8 show that the computed vibrational energies for SiO$^+$ and TaS molecules are highly consistent with experimental RKR data^40^ throughout all electronic states. This consistency is supported by the fractional formalism. For SiO$^+$, the fractional case (49)) achieves close agreement with the RKR values for the ${B^2\Sigma ^+}$ state, yielding a MAPD of 0.0082$\%$. The fractional model confirms the extraordinary precision of the TaS molecule, with MAPD values as low as 0.0380$\%$ for the ${A^2\Delta }$ state and 0.0421$\%$ for the ${B^2\Delta }$ state.

In Table 9, we report the vibrational energies for the ${X^1\Sigma ^+}$, ${D^1\Pi }$ and ${E^1\Sigma ^+}$ states of the SiS molecule. Our findings agree closely with the benchmark RKR data^41^. The small MAPD strongly confirms this conclusion. The ground ${X^1\Sigma ^+}$ state exhibits close agreement, with MAPDs of only 0.0671$\%$ and 0.0538$\%$ for Eqs. (52)) and (49)), respectively. The ${D^1\Pi }$ state has strong consistency, with the fractional model (Eq. 49)) showing even better agreement with a MAPD of 0.2504$\%$. The fractional model (MAPD = 1.6697$\%$) is more consistent with the RKR data than the traditional procedure, though deviations are noticeable in the high-lying ${E^1\Sigma ^+}$ state. This consistent behavior across multiple electronic states illustrates that our techniques, particularly the fractional formalism of Eq. 49), provide a reliable representation of the vibrational structure. This tendency is also verified for the ZrS molecule since, in comparison to the ordinary situation, the fractional model (Eq. 49)) yields the minimal MAPD for both the ${X^1\Sigma ^+}$ state (0.0362$\%$) and the ${B^1\Pi }$ state (0.0245$\%$), as seen in Table 10.

Furthermore, the results for the TaO, AlH, and CaH molecules in Tables 11 and 12 confirm definitively that the fractional model is more accurate and consistent. The fractional case (Eq. 49)) provides the TaO molecule the lowest MAPD for both the ${X^2\Delta _{3/2}}$ state (0.1582$\%$) and the ${K^2\Phi _{5/2}}$ state (0.1220$\%$). This indicates that it is better than the traditional case. Similarly, the fractional formalism produces a minimal MAPD of 0.0325$\%$ for the AlH (${X^1\Sigma ^+}$) molecule and an enhanced MAPD of 0.0454$\%$ for the CaH (${X^2\Sigma ^+}$) molecule, as indicated in Table 12. In this investigation of several molecular systems, the fractional case (Eq. 49)) consistently yields more accurate vibrational energies. This reveals that it is an appropriate method for characterizing the RKR potential curves of diverse diatomic molecules. To assess the reliability of the analytical solutions, the vibrational energies of the CS (${X^1\Sigma ^+}$) and CN (${X^2\Sigma ^+}$) molecules were calculated and listed in Tables 13 and 14. These results were then compared with those of Ref.^50^, where the Schrödinger equation was solved numerically using the Numerov method for the Morse potential (MP), Frost-Musulin potential (FMP), and Poschl-Teller potential (PTP). The comparison indicates that the vibrational energies predicted by our classical model (Eq. 52) are in satisfactory agreement with both the numerical results of Ref.^50^. and the experimental RKR data^51^.

To analyze the behavior of absolute percentage deviations for vibrational energies obtained from both classical and fractional models, we included plots illustrating the level-by-level errors of the pure vibrational energies across the entire range of experimentally available bound states of the diatomic molecules considered in this study (**See Supplementary Figs.** **A1-A6**).

**Table 8:** Vibrational energies (${cm^{-1}}$) for the (${X^2\Delta }$), (${A^2\Delta }$) and (${B^2\Delta }$) states of TaS molecule.

*n*	${X^2\Delta }$			${A^2\Delta }$			${B^2\Delta }$		
	RKR^40^	Eq. (52)	Eq. (49)	RKR^40^	Eq. (52)	Eq. (49)	RKR^40^	Eq. (52)	Eq. (49)
0	275.6	276.5	276.4	265.3	266.1	265.9	257.0	257.9	257.5
1	824.8	825.7	825.3	793.9	794.9	794.1	769.2	770.3	769.2
2	1371.4	1372.2	1371.6	1319.8	1321.1	1319.8	1278.8	1280.4	1278.5
3	1915.0	1916.0	1915.1	1843.1	1844.7	1843.0	1785.8	1788.0	1785.4
4	2456.0	2457.0	2455.8	2363.7	2365.9	2363.6	2290.1	2293.3	2290.0
5	2994.3	2995.3	2993.9	2881.7	2884.5	2881.7	2791.9	2796.2	2792.1
6	3529.8	3530.9	3529.2	3397.0	3400.5	3397.3	3291.0	3296.7	3291.9
7	4062.5	4063.7	4061.7						
MAPD%		0.0858	0.0510		0.1312	0.0380		0.1739	0.0421

**Table 9:** Vibrational energies (${cm^{-1}}$) for the (${X^1\Sigma ^+}$), (${D^1\Pi }$) and (${E^1\Sigma ^+}$) states of SiS molecule.

*n*	${X^1\Sigma ^+}$			${D^1\Pi }$			${E^1\Sigma ^+}$		
	RKR^41^	Eq. (52)	Eq. (49)	RKR^41^	Eq. (52)	Eq. (49)	RKR^41^	Eq. (52)	Eq. (49)
0	374.2	374.2	374.3	255.4	255.9	254.9	202.4	202.4	199.1
1	1119.0	1118.7	1119.1	763.3	763.2	760.1	603.9	604.8	595.0
2	1858.3	1858.0	1858.8	1264.3	1265.7	1260.5	1000.5	1004.0	987.7
3	2592.0	2592.2	2593.2	1759.9	1763.4	1756.2	1395.4	1400.0	1377.4
4	3321.8	3321.23	3322.5	2249.0	2256.3	2247.3	1788.2	1792.8	1764.0
5	4045.9	4045.1	4046.7	2732.2	2744.5	2733.5	2173.1	2182.4	2147.4
6	4763.5	4763.8	4765.7	3215.1	3228.0	3215.1	2558.9	2568.8	2527.8
7	5476.9	5477.4	5479.5	3687.2	3706.7	3692.0	2935.9	2952.0	2905.1
8	6181.6	6185.8	6188.2	4152.5	4180.6	4164.1	3311.2	3332.0	3279.3
9	6886.8	6889.1	6891.8	4610.8	4649.7	4631.5	3680.7	3708.8	3650.4
10	7587.5	7587.2	7590.1	5061.8	5114.2	5094.2	4050.0	4082.4	4018.4
11	8276.4	1118.7	1119.1				4410.2	4452.8	4383.3
12	8969.0	8967.9	8971.4				4765.3	4820.0	4745.1
13	9651.9	9650.6	9654.3				5115.1	5184.0	5103.8
14	10329.8	10328.1	10332.0				5459.4	5544.9	5459.4
15	11002.5	11000.4	11004.6				5798.1	5902.5	5811.9
16	11670.1	11667.6	11672.0				6131.0	6256.9	6161.3
17	12332.6	12329.6	12334.2				6457.9	6608.1	6507.7
18	12990.0	12986.5	12991.3				6778.7	6956.1	6850.9
19	13642.3	13638.2	13643.3				7093.2	7300.9	7191.0
20	14289.5	14284.8	14290.1				7401.2	7642.5	7528.1
21	14931.7	14926.2	14931.7				7702.5	7980.9	7862.0
22	15568.7	15562.4	15568.2				7997.1	8316.1	8192.8
23	16200.7	16193.5	16199.5				8284.6	8648.1	8520.6
24	16827.6	16819.5	16825.6				8565.0	8976.9	8845.2
25	17440.8	17440.3	17446.6				8838.1	9302.5	9166.8
26	18066.2	18055.9	18062.5				9103.7	9624.9	9485.3
27	18677.8	18666.4	18673.1				9361.7	9944.1	9800.6
28	19284.4	19271.7	19278.6				9611.9	10260.0	10113.0
29	19885.9	19871.9	19879.0						
30	20482.5	20466.9	20474.2						
31	21073.9	21056.8	21064.3						
32	21660.3	21641.5	21649.1						
33	22241.6	22221.1	22228.9						
34	22817.9	22795.5	22803.4						
35	23389.1	23364.8	23372.8						
36	23955.3	23928.9	23937.1						
37	24516.4	24487.8	24496.2						
38	25072.5	25041.6	25050.1						
39	25623.6	25590.2	25598.9						
40	26169.7	26133.7	26142.5						
41	26710.7	26672.0	26681.0						
42	27246.7	27205.2	27214.3						
43	27777.7	27733.2	27742.4						
44	28303.7	28256.1	28265.4						
45	28824.6	28773.8	28783.2						
46	29340.6	29286.4	29295.9						
47	29851.5	29793.8	29803.4						
MAPD%		0.0671	0.0538		0.4359	0.2504		2.2553	1.6697

For CaH $(X^2\Sigma ^+)$, NaK $(c^3\Sigma ^+)$, and RbH $(X^1\Sigma ^+)$ molecules, the classical curves increase monotonically with the vibrational quantum number (*n*), indicating that the classical model becomes progressively less accurate at higher vibrational levels, whereas the fractional curves initially decrease, reach a minimum at intermediate *n*, and then rise more slowly than the classical ones, yielding smaller errors across most of the plotted range. The absolute percentage deviations for the Na$_2$ $(c^1\Pi _u)$ molecule decrease as the vibrational quantum number increases, subsequently remaining minimal and stable at higher vibrational levels, particularly within the fractional model. Similar trends were observed for the diatomic molecules SiS $(X^1\Sigma ^+)$, SiS $(D^1\Pi )$, SiS $(E^1\Sigma ^+)$, and SiC $(X^3\Pi )$, with the classical limit indicating lower deviations at smaller *n*, whereas the fractional framework reduces deviations as the quantum number increases. For different electronic states of the TaS molecule, the deviations consistently decrease as the quantum number increases, with the smallest errors observed in the energy estimates determined from the fractional derivative formalism. In the case of the CO $(X^1\Sigma ^+)$ molecule, both models exhibit small deviations at low and moderate *n*, and the fractional curve lies below the classical one, indicating slightly better agreement with the RKR data. At higher vibrational levels $(n \ge 25)$, the errors for both models increase monotonically. However, the classical and fractional curves remain close, showing comparable accuracy in the highly excited states. Comparable deviations were observed for the other molecules investigated in this study. The deviations of the vibrational energy obtained from the fractional derivative and traditional Schrödinger equation with the Morse potential at higher quantum levels may be ascribed to anharmonic effects and spectroscopic parameters of the molecules. Diatomic molecules with large reduced masses and equilibrium bond lengths tend to possess larger rotational inertia, while lighter molecules have smaller rotational inertia and higher centrifugal distortions, causing their bond to stretch. Generally, the fractional derivative method diminishes the deviations of the vibrational energy relative to the classical limit, as demonstrated by the calculated MAPD values and the level-by-level error plots.

**Table 10:** Vibrational energies for (${cm^{-1}}$), (${X^1\Sigma ^+}$) and (${B^1\Pi }$) states of ZrS molecule.

*n*	${X^1\Sigma ^+}$			${B^1\Pi }$		
	RKR^40^	Eq. (52)	Eq. (49)	RKR^40^	Eq. (52)	Eq. (49)
0	273.8	273.8	274.0	247.6	248.0	247.9
1	819.2	819.0	819.5	740.9	741.3	741.1
2	1361.6	1361.0	1361.9	1231.6	1232.0	1231.8
3	1901.1	1899.8	1901.1	1719.8	1720.2	1719.8
4	2437.7	2435.5	2437.1	2205.3	2205.8	2205.3
5	2971.4	2968.0	2970.0	2688.2	2688.7	2688.1
6	3502.0	3497.4	3499.7	3168.5	3169.1	3168.4
MAPD%		0.0693	0.0362		0.0456	0.0245

**Table 11:** Vibrational energies (${cm^{-1}}$) for (${X^2\Delta _{3/2}}$) and (${K^2\Phi _{5/2}}$) states of TaO molecule.

	${X^2\Delta _{3/2}}$			${K^2\Phi _{5/2}}$		
*n*	RKR^40^	Eq. (52)	Eq. (49)	RKR^40^	Eq. (52)	Eq. (49)
0	513.5	515.9	516.3	451.8	456.5	454.5
1	1535.2	1536.7	1537.9	1349.9	1355.7	1349.9
2	2549.8	2549.6	2551.6	2240.6	2248.8	2239.1
3	3557.2	3554.5	3557.2	3124.0	3135.6	3122.2
4	4557.3	4551.3	4554.8	4000.1	4016.2	3999.1
5	5550.4	5540.2	5544.4	4868.8	4890.6	4869.8
6	6536.2	6521.0	6525.9	5730.1	5758.8	5734.4
MAPD%		0.1705	0.1582		0.5082	0.1220

**Table 12:** Calculated energies (${cm^{-1}}$) for AlH (${X^1\Sigma ^+}$) and CaH (${X^2\Sigma ^+}$) molecules.

*n*	AlH (${X^1\Sigma ^+}$)			CaH (${X^2\Sigma ^+}$)		
	RKR^42^	Eq. (52)	Eq. (49)	RKR^42^	Eq. (52)	Eq. (49)
0	834.04	834.2	834.0	644.39	644.5	643.9
1	2459.19	2460.2	2459.7	1904.53	1905.5	1903.8
2	4028.31	4029.5	4028.7	3126.47	3129.3	3126.5
3	5542.84	5542.3	5541.2	4310.20	4315.6	4311.9
4	7004.21	6998.4	6997.0	5455.74	5464.7	5460.0
MAPD%		0.0369	0.0325		0.0903	0.0454

**Table 13:** Vibrational energies (${cm^{-1}}$) for CS (${X^1\Sigma ^+}$) molecule.

*n*	RKR^51^	MP^50^	FMP^50^	PTP^50^	Eq. 52	Eq. 49
0	640.9	640.79	640.84	640.81	640.8061	641.6995
1	1913.1	1911.91	1911.95	1911.93	1911.977	1914.628
2	3172.3	3169.05	3169.10	3169.07	3169.227	3173.597
3	4418.6	4412.23	4412.30	4412.25	4412.555	4418.605
4	5652.0	5641.45	5641.55	5641.47	5641.963	5649.652
5	6872.5	6856.69	6856.86	6856.71	6857.448	6866.74
6	8080.1	8057.97	8058.23	8057.99	8059.013	8069.867
7	9274.7	9245.29	9245.68	9245.31	9246.656	9259.034
MAPD%		0.1667	0.1636	0.1659	0.1584	0.0833

**Table 14:** Vibrational energies (${cm^{-1}}$) for CN (${X^2\Sigma ^+}$) molecule.

*n*	RKR^51^	MP^50^	FMP^50^	PTP^50^	Eq. 52	Eq. 49
0	1031.0	1030.04	1030.15	1030.08	1030.06	1037.22
1	3073.4	3064.57	3064.80	3064.61	3064.7	3085.82
2	5089.7	5065.03	5065.54	5065.07	5065.38	5099.98
3	7079.7	7031.44	7032.37	7031.48	7032.09	7079.7
4	9043.6	8963.79	8965.32	8963.83	8964.84	9024.98
5	10,981.3	10,862.07	10,864.41	10,862.11	10,863.6	10,935.8
6	12,892.9	12,726.29	12,729.65	12,726.33	12,728.4	12,812.2
7	14,778.2	14,556.45	14,561.06	14,556.49	14,559.3	14,654.2
MAPD%		0.7885	0.7713	0.7874	0.7780	0.4118

**Table 15:** Rovibrational energy levels (in eV) for the CO (${X^1\Sigma ^+}$) molecule.

*n*	*J*	Energy (eV)	
		Eq. 52	Ref.^8^
0	0	− 11.09153379	− 11.09153532
	1	− 11.09105722	− 11.09105875
	2	− 11.09010408	− 11.09010565
1	0	− 10.82581753	− 10.82582206
	1	− 10.82534503	− 10.82534959
	2	− 10.82440005	− 10.82440465
2	0	− 10.56332281	− 10.56333028
	1	− 10.56285438	− 10.56286190
	2	− 10.56191755	− 10.56192516
0	10	− 11.06533119	− 11.06533330
3		− 10.27851923	− 10.27853420
5		− 9.77008565	− 9.77011230
0	20	− 10.99158602	− 10.99159010
3		− 10.20666789	− 10.20669750
5		− 9.69949686	− 9.69955630
0	25	− 10.93696577	− 10.93697160
3		− 10.15345224	− 10.15349400
5		− 9.64721760	− 9.64730340

As a further examination of the accuracy of the expressions obtained via the GFNU method and Pekeris-type approximation, we have computed the ro-vibrational energies of the CO (${X^1\Sigma ^+}$) molecule for several high-lying states ($J = 10, 20, 25; n = 0, 1, 2, 5$) for the shifted Morse potential, and we have compared our results with those reported in Ref.^8^. Table 15 demonstrates that the rovibrational energies computed via the analytical GFNU method employing the Pekeris-type approximation (Eq. 52)) are in good agreement with the high-precision numerical benchmark obtained through the generalized pseudospectral technique^8^. The near-exact correspondence demonstrates that the derived formulas are precise and robust for high rotational quantum numbers up to $J=25$. This result demonstrates that the Pekeris approximation remains highly effective within the context of the shifted Morse potential framework. It maintains the accuracy of spectroscopy while providing a rapid and straightforward analysis for a broad spectrum of rotational and vibrational states.

## Conclusion

This study successfully demonstrated how the generalized fractional derivative framework significantly improves the ability to model the vibrational energy spectra of diatomic molecules. The derivation of analytical solutions to the *D*-dimensional SE has been facilitated by the development of the GFNU method, which has provided a diverse and effective tool for quantum mechanical analysis. The principal finding of this investigation is that the fractional parameter $\rho$ has a significant influence on the energy spectra of diatomic molecules. This provides a further dimension of variability that allows the model to fit experimental data more precisely. The proper application of the Pekeris approximation on the centrifugal term confirmed that our solutions for rotating molecules in various electronic states were reliable. The Morse potential produces reliable fits for a diverse set of twenty-two diatomic molecules, consistently when compared to experimental RKR data. As demonstrated by the MAPD values, the vibrational energies determined from the fractional model (Eq. 49)) using the fitted fractional parameters consistently outperformed those obtained from the classical model (Eq. 52)). Based on the analysis of absolute percentage deviations in level-by-level error plots for all examined diatomic molecules, the fractional derivative case yields smaller vibrational energy errors compared to the classical limit as the quantum number increases. This work confirms that the generalized fractional derivative framework is an effective and reliable technique for spectroscopic modelling. It provides a robust mathematical framework that integrates traditional quantum mechanical models with accurate experimental results in molecular physics and quantum chemistry. Further studies could explore the utilization of this GFNU method with alternative empirical potentials and more complex molecular systems^33,34,43^.

## Supplementary Information

Supplementary Information.

## Data Availability

All data generated or analysed during this study are available upon reasonable request from the corresponding author.

## References

[1] Abu-Shady, M. & Khokha, E. M. A precise estimation for vibrational energies of diatomic molecules using the improved Rosen-Morse potential. *Sci. Rep.* **13**, 11578 (2023). 37463934 10.1038/s41598-023-37888-2 PMC10354199

[2] Abu-Shady, M., Khokha, E. M., & Abdel-Karim, T. A. The generalized fractional NU method for diatomic molecules in the Deng–Fan model. *Eur. Phys. J. D* **76**, 159 (2022). 10.1140/epjd/s10053-022-00480-w PMC9449962 36091719

[3] Abu-Shady, M. & Khokha, E. M. On prediction of the fractional vibrational energies for diatomic molecules with the improved Tietz potential. *Mol. Phys.* **120**(24), e2140720 (2022).

[4] Abu-Shady, M. & Khokha, E. M. Bound state solutions of the Dirac equation for the generalized Cornell potential model. *Int. J. Mod. Phys. A* **36**(29), 2150195 (2021).

[5] Khokha, E. M., Abu-Shady, M. & Abdel-Karim, T. A. The influence of magnetic and Aharanov-Bohm fields on energy spectra of diatomic molecules in the framework of the Dirac equation with the generalized interaction potential. *Int. J. Quantum Chem.* **123**(4), e27031 (2023).

[6] Morse, P. M. Diatomic molecules according to the wave mechanics. II. Vibrational levels. *Phys. Rev.* **34**, 57–64 (1929).

[7] Shui, Z. W. & Jia, C. S. Relativistic rotation–vibrational energies for the 107Ag 109Ag isotope. *Eur. Phys. J. Plus* **132**, 292 (2017).

[8] Roy, A. K. Accurate ro-vibrational spectroscopy of diatomic molecules in a Morse oscillator potential. *Res. Phys.* **3**, 103–108 (2013).

[9] Zuniga, J., Bastida, A., & Requena, A. An analytical perturbation treatment of the rotating Morse oscillator. *J. Phys. B Atom. Mol. Opt. Phys.* **41**, 105102 (2008).

[10] Okorie, U. S. & Rampho, G. J. Theoretical computation of thermodynamic functions of sodium dimer with modified shifted Morse potential. *Comput. Theor. Chem.* **1241**, 114925 (2024).

[11] Berkdemir, C. Pseudospin symmetry in the relativistic Morse potential including the spin–orbit coupling term. *Nucl. Phys. A* **770**, 32–39 (2006).

[12] Njoku, I. J. Relativistic solutions of the Morse potential via the formula method. *Chem. Phys. Impact* **5**, 100113 (2022).

[13] Bayrak, O. & Boztosun, I. Arbitrary l-state solutions of the rotating Morse potential by the asymptotic iteration method. *J. Phys. A: Math. Gen.* **39**, 6955–6963 (2006).

[14] Berkdemir, C. & Han, J. Any l-state solutions of the Morse potential through the Pekeris approximation and Nikiforov-Uvarov method. *Chem. Phys. Lett.* **409**, 203–207 (2005).

[15] Soylu, A., Bayrak, O. & Boztosun, I. Effect of the velocity-dependent potentials on the energy eigenvalues of the Morse potential. *Cent. Eur. J. Phys.* **10**(4), 953–959 (2012).

[16] Selg, M. & Belous, V. Reference potential approach to the energy eigenvalue problem of a rotating diatomic molecule. *Chem. Phys. Lett.* **462**, 337–343 (2008).

[17] Mirzanejad, A. & Varganov, S. A. Derivation of Morse potential function. *Mol. Phys.* **123**(3), e2360542 (2024).

[18] Al-Dossary, O. M. Morse potential eigen-energies through the asymptotic iteration method. *Int. J. Quantum Chem.* **107**, 2040–2046 (2007).

[19] Sharma, A. & Sastri, O. S. K. S. Numerical solution of Schrödinger equation for rotating Morse potential using matrix methods with Fourier sine basis and optimization using variational Monte Carlo approach. *Int. J. Quantum Chem.* **121**, e26682 (2021).

[20] Sastri, O. S. K. S. et al. Simulation of vibrational spectrum of diatomic molecules using Morse potential by matrix methods in Gnumeric worksheet. Phys. Educ. **36**, 1 (2020).

[21] Ibrahim, A., Fedoul, A., Janati Idrissi, M., Ababou, Y., & Sayouri, S. Analytical development to determine vibrational energy levels and dissociation energy of diatomic molecules. *FirePhysChem* (2024).

[22] Amila, I., Fedoul, A., Janati Idrissi, M., Chatwiti, A., & Sayouri, S. An innovative treatment of anharmonic and Morse potentials to determine the spectroscopic constants of diatomic molecules. *Phys. Scripta* **99**(7), 075413 (2024).

[23] Janati Idrissi, M., Fedoul, A., Amila, I., Chatwiti, A. & Sayouri, S. A new analytical approach to study the anharmonic and Morse potentials of diatomic molecules. *Int. J. Nanosci. Nanotechnol.* **19**(3), 165–172 (2023).

[24] Onate, C. A. et al. Analytical solutions and Herzberg’s energy level for modified shifted Morse molecular system. *Heliyon* **9**, e13526 (2023). 36825167 10.1016/j.heliyon.2023.e13526 PMC9941989

[25] Qiang, W. C. & Dong, S.-H. Arbitrary l-state solutions of the rotating Morse potential through the exact quantization rule method. *Phys. Lett. A* **363**, 169–176 (2007).

[26] Chenaghlou, A., Aghaei, S. & Niari, N. G. The solution of D+1-dimensional Dirac equation for diatomic molecules with the Morse potential. *Eur. Phys. J. D* **75**, 139 (2021).

[27] Du, J. F., Guo, P. & Jia, C.-S. D-dimensional energies for scandium monoiodide. *J. Math. Chem.* **52**, 2559–2569 (2014).

[28] Onate, C. A. et al. Non-relativistic molecular modified shifted Morse potential system. *Sci. Rep.* **12**, 1588 (2022). 36071068 10.1038/s41598-022-19179-4 PMC9452569

[29] Sharma, A. & Sastri, O. S. K. S. Numerical simulation of ro-vibrational spectra for diatomic molecules using the Numerov matrix method. *Eur. J. Phys.* **43**, 015404 (2022).

[30] Bayrak, O., Soylu, A. & Boztosun, I. The relativistic treatment of spin-0 particles under the rotating Morse oscillator. *J. Math. Phys.* **51**, 112301 (2010).

[31] Xie, X. J. & Jia, C. S. Solutions of the Klein-Gordon equation with the Morse potential energy model in higher spatial dimensions. *Phys. Scr.* **90**, 035207 (2015).

[32] Abu-Shady, M., & Kaabar, M. K. A. A generalized fractional derivative and its applications to fractional differential equations. Math. Probl. Eng. Article ID 9444803 (2021).

[33] Abu-Shady, M., Abdel-Karim, T. A. & Khokha, E. M. Binding Energies and Dissociation Temperatures of Heavy Quarkonia at Finite Temperature and Chemical Potential in the N-Dimensional Space. *Adv. High Energy Phys.* **2018**, 7356843 (2018).

[34] Abu-Shady, M. & Khokha, E. M. Heavy-Light Mesons in the Nonrelativistic Quark Model Using Laplace Transformation Method. *Adv. High Energy Phys.* **2018**, 7032041 (2018).

[35] Ferber, R. et al. The c, b, and a states of NaK revisited. *J. Chem. Phys.* **112**(13), 5740–5750 (2000).

[36] Jastrzebski, W., Kowalczyk, P., & Pashov, A. The C state of Na molecule studied by polarization labelling spectroscopy method. Spectrochim. *Acta Part A Mol. Biomol. Spectrosc.* **57**(9), 1829–1831 (2001). 10.1016/s1386-1425(01)00405-x 11506033

[37] Stwalley, W. C., Zemke, W. T. & Yang, S. C. Spectroscopy and structure of the alkali hydride diatomic molecules and their ions. *J. Phys. Chem. Ref. Data* **20**(1), 153–187 (1991).

[38] Reddy, R. R., Rao, T. V. R. & Viswanath, R. Potential energy curves and dissociation energies of NbO, SiC, CP, PH, SiF, and NH. *Astrophys. Space Sci.* **189**(1), 29–38 (1992).

[39] Kirschner, S. M. & Watson, J. K. G. Second-order semiclassical calculations for diatomic molecules. *J. Mol. Spectrosc.* **51**(2), 321–333 (1974).

[40] Reddy, R. R. et al. Spectroscopic studies on astrophysically interesting TaO, TaS, ZrS, and SiO molecules. *Astrophys. Space Sci.* **281**(4), 729–741 (2002).

[41] Lakshman, S. J., Rao, T. V. R. & Daidu, G. T. The true potential energy curves for different states of SiO and SiS molecules. *Pramana* **7**(6), 369–377 (1976).

[42] Narasimhamurthy, B. & Rajamanickam, N. Astrophysical molecules of AlH and CaH: RKR potential and dissociation energies. *J. Astrophys. Astron.* **4**(1), 53–58 (1983).

[43] Lippincott, E. R. A new relation between potential energy and internuclear distance. *J. Chem. Phys.* **21**(11), 2070–2071 (1953).

[44] Okorie, U. S. et al. Diatomic molecules energy spectra for the generalized Mobius square potential model. *Int. J. Mod. Phys. B* **34**(21), 2050209 (2020). 10.1007/s00894-020-04449-7 32621032

[45] Eyube, E. S. Reparametrised Pöschl-Teller oscillator and analytical molar entropy equation for diatomic molecules. *Mol. Phys.* **120**(8), e2037774 (2022).

[46] Eyube, E. S., Notani, P. P. & Dikko, A. B. Modeling of diatomic molecules with modified hyperbolical-type potential. *Eur. Phys. J. Plus* **137**(3), 329 (2022).

[47] Yanar, H. et al. Ro-vibrational energies of CO molecule via improved generalized Pöschl-Teller potential and Pekeris-type approximation. *Eur. Phys. J. Plus* **135**(3), 292 (2020).

[48] Omugbe, E. et al. Non-relativistic energy equations for diatomic molecules constrained in a deformed hyperbolic potential function. *J. Mol. Model.* **30**(3), 74 (2024). 38374405 10.1007/s00894-024-05855-x

[49] Liu, J.-Z. & Jia, C.-S. Prediction of vibrational energy levels for the CO molecule and Li dimer. *Chem. Phys. Lett.* **803**, 139791 (2022).

[50] Rasoolzadeh, M. & Islampour, R. Estimation of vibrational energy levels of diatomic molecules (CN, CO and CS) using Numerov algorithm and comparison with the empirical values. *Aust. J. Basic Appl. Sci.* **5**(12), 2041–2047 (2011).

[51] Reddy, R. R., Ahammed, Y. N., Gopal, K. R. & Basha, D. B. Intercomparison of molecular collision integral data for high-temperature air species. *Astrophys. Space Sci.* **286**(3), 419–436 (2003).

