# Supplementary material for: Improved modelling for vibrational energies of diatomic molecules using the generalized fractional derivative
Source: Sci Rep. 2026 Apr 9;16:12037. doi: 10.1038/s41598-026-39091-5 (PMC13069049; doi:10.1038/s41598-026-39091-5)
Supplement: Supplementary file 1 — Supplementary Information. [file 41598_2026_39091_MOESM1_ESM.pdf]

Improved Modelling for Vibrational Energies of Diatomic Molecules Using the Generalized Fractional Derivative

E. M. Khokha [1,*], M. Abu-Shady [2], E. Omugbe [3] and N. S. Sweed [4]

[1] Faculty of Computer Science and Engineering, King Salman International University (KSIU), South Sinai, El Tur 46511, Egypt (Corresponding author: emad.khokha@ksiu.edu.eg)

[2] Department of Mathematics and Computer Science, Faculty of Science, Menoufia University, Shibin El Kom 46511, Egypt (dr.abushady@gmail.com)

[3] Department of Physics and Engineering Physics, Faculty of Science, Obafemi Awolowo University, Ile-Ife, Osun State, Nigeria (omugbeekwevugbe@gmail.com)

[4] Department of Basic Science, Modern Academy for Engineering and Technology, Cairo 11439, Egypt (naglaa.sayed@eng.modern-academy.edu.eg)

A Appendix

The absolute percentage error of the Morse potential energies from the experimental RKR data points for diatomic molecule is defined by:

$$\text{Absolute error } \% = \left| \frac{E_{RKR} - E_{RKR}}{E_{theo.}} \right| \times 100, \tag{A.1}$$

where E_{RKR} denotes the experimental RKR data point, and $E_{theo.}$ are the fractional and classical vibrational energy eigenvalues determined by Eq. (49) and Eq. (58) respectively.

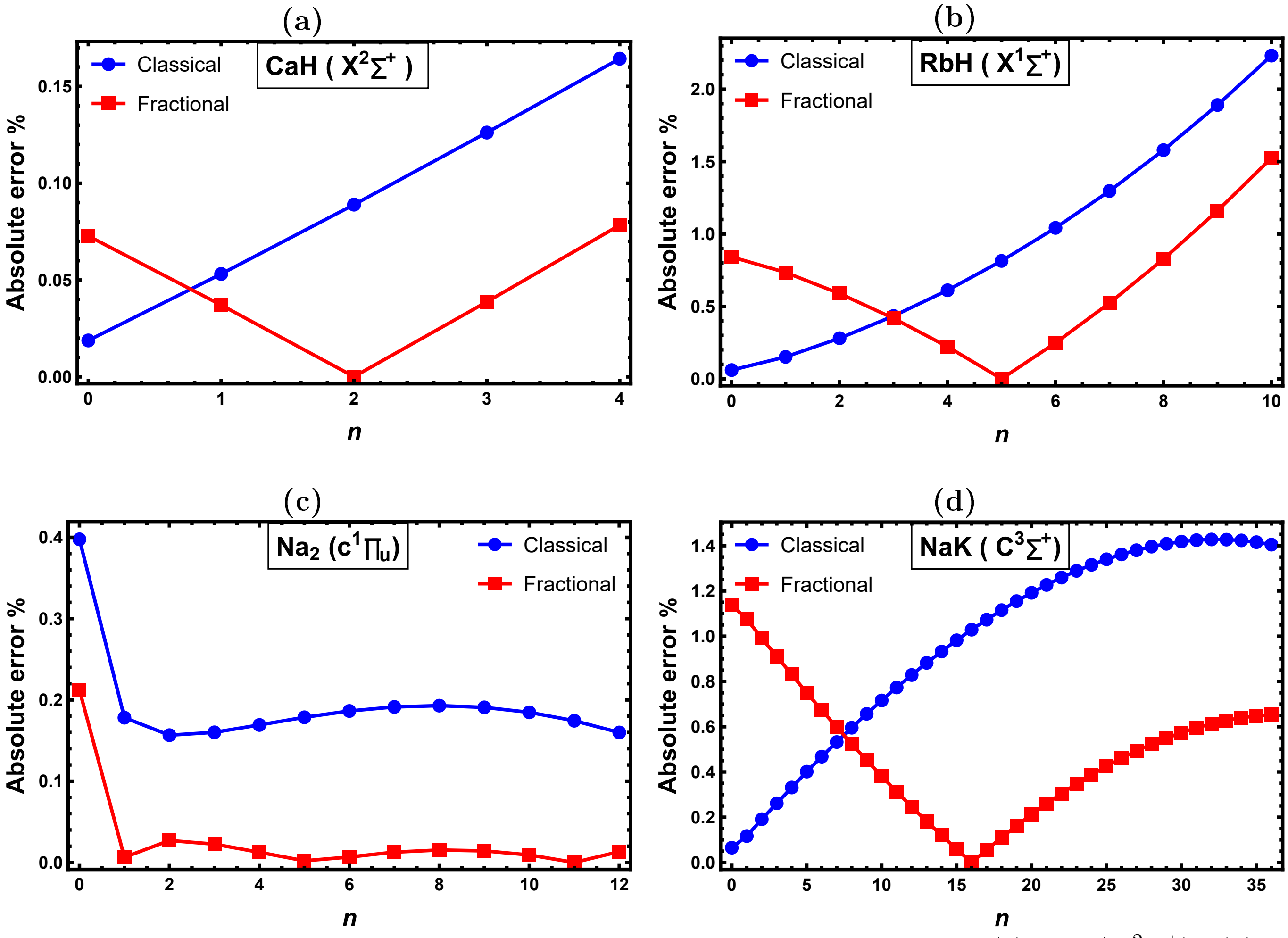

Fig. A1. Absolute percentage deviations of vibrational energies for the: (a) CaH($X^2\Sigma^+$), (b) RbH($X^1\Sigma^+$), (c) $Na_2(c^1\Pi_u)$ and (d) NaK($c^3\Sigma^+$).

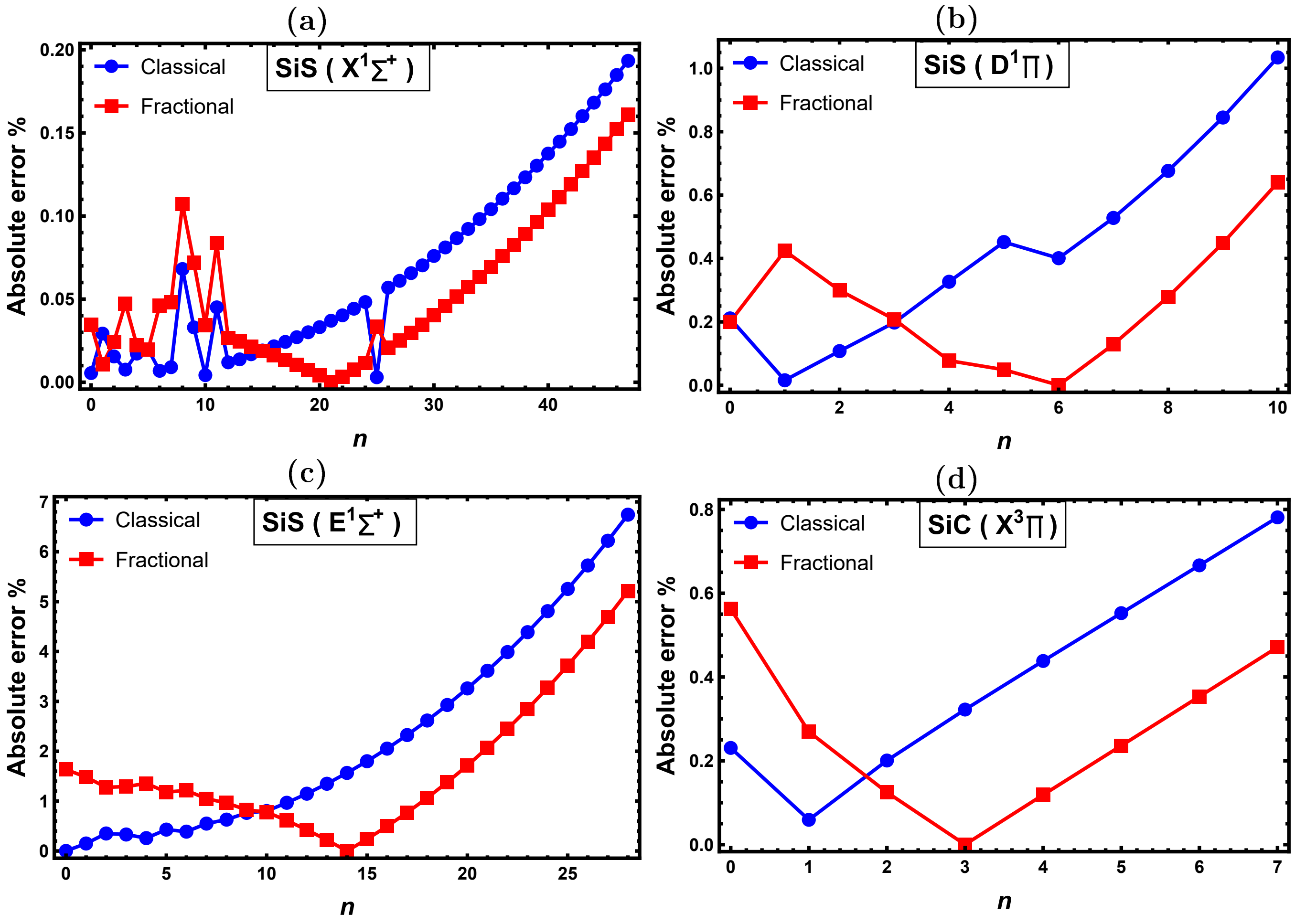

Fig. A2. Absolute percentage deviations of vibrational energies for the: (a) SiS($X^1\Sigma^+$), (b) SiS($D^1\Pi$), (c) SiS($E^1\Sigma^+$) and (d) SiC($X^3\Pi$).

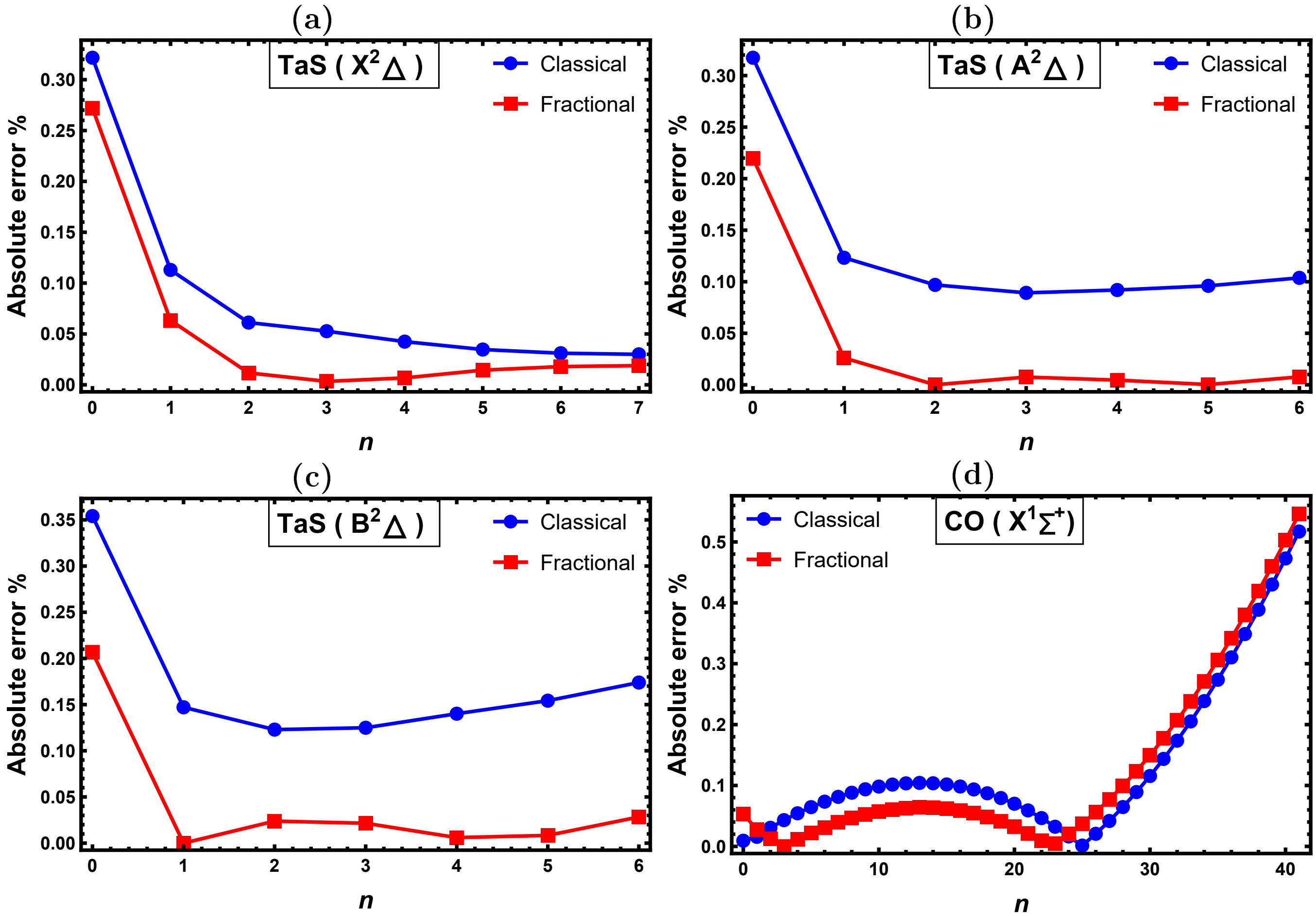

Fig. A3. Absolute percentage deviations of vibrational energies for the: (a) TaS($X^2\Delta$), (b) TaS($A^2\Delta$), (c) TaS($B^2\Delta$) and (d) CO($X^1\Sigma^+$).

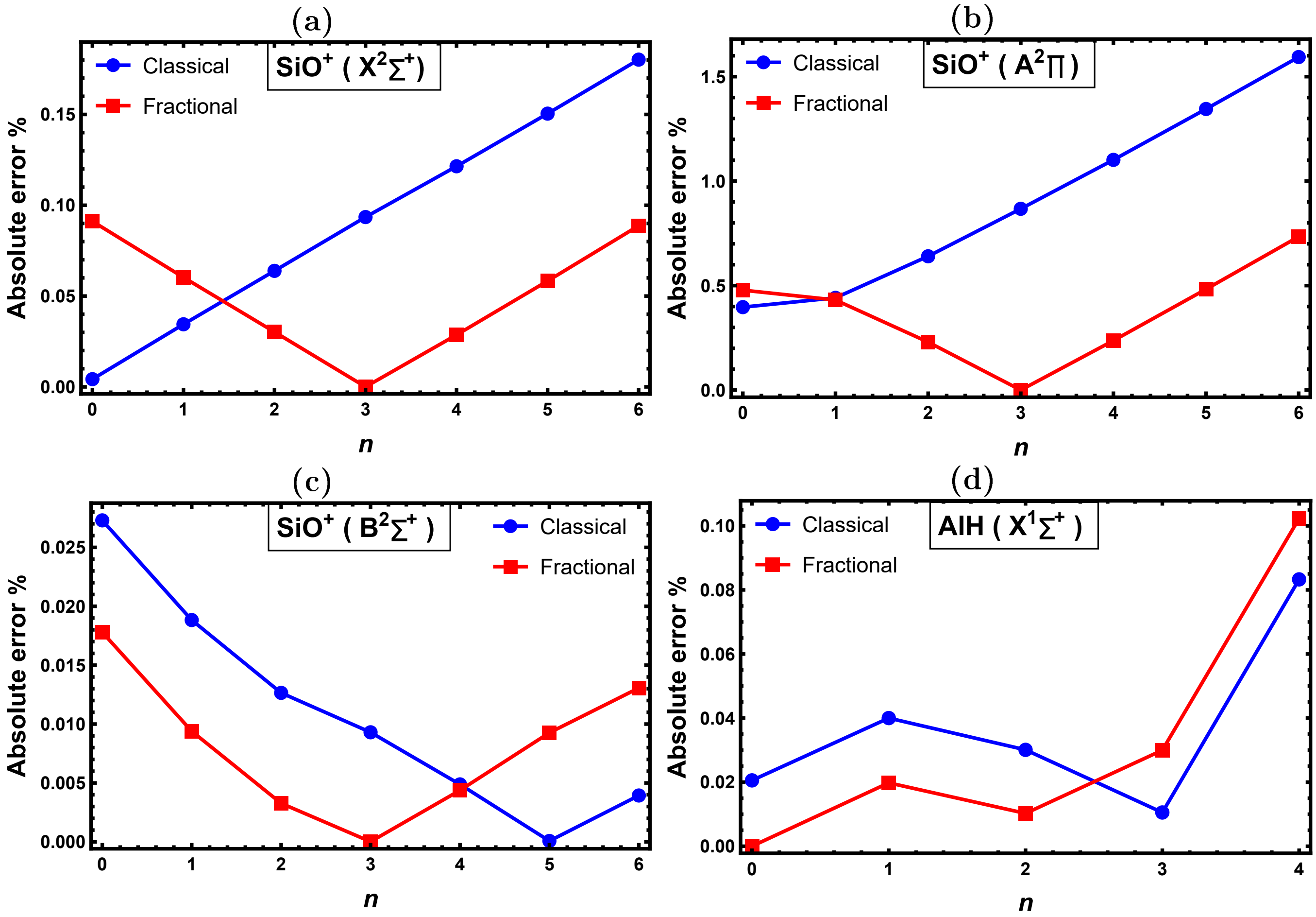

Fig. A4. Absolute percentage deviations of vibrational energies for the: (a) $SiO^+(X^2\Sigma^+)$, (b) $SiO^+(A^2\Pi)$, (c) $SiO^+(B^2\Sigma^+)$ and (d) $AlH(X^1\Sigma^+)$.

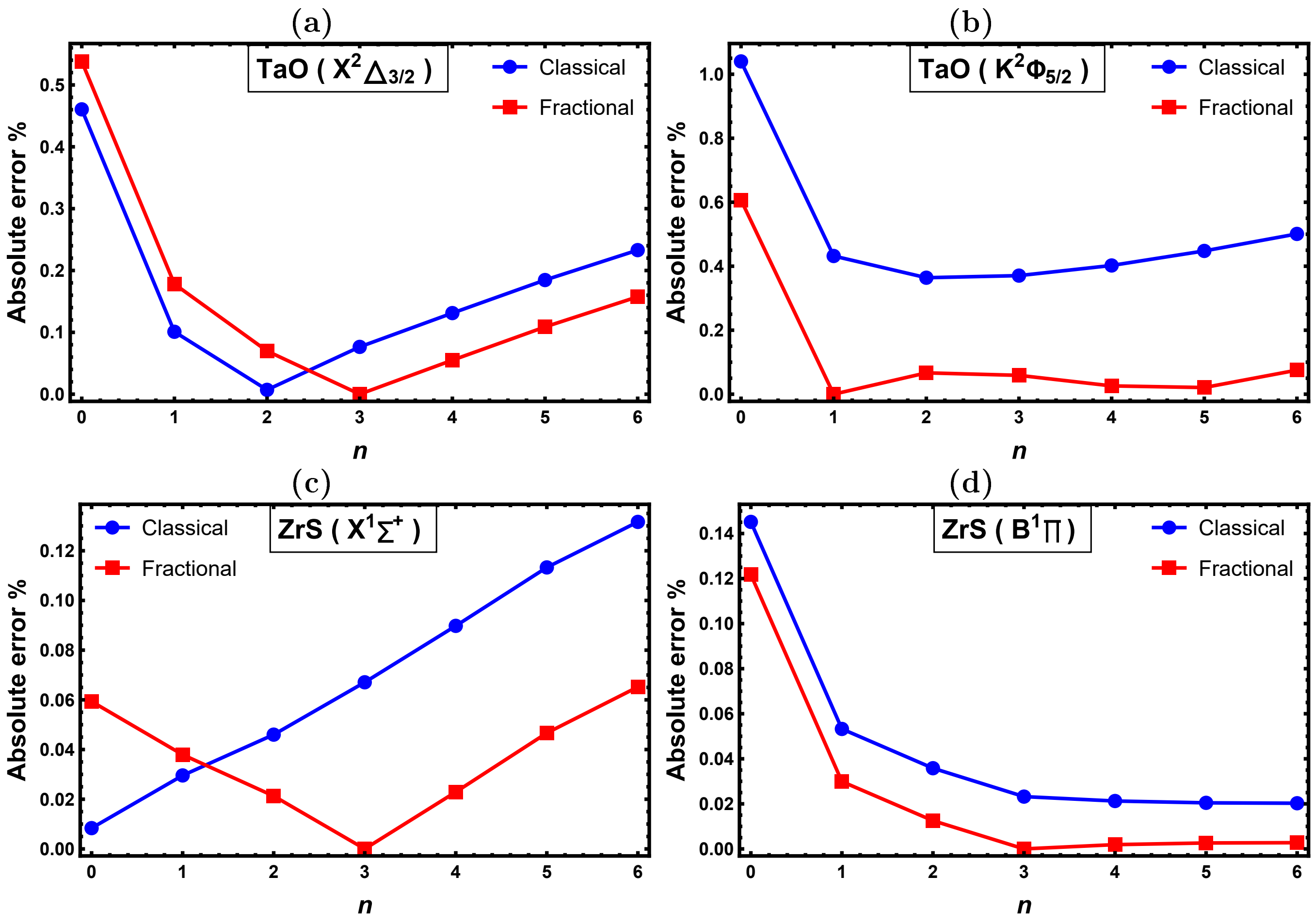

Fig. A5. Absolute percentage deviations of vibrational energies for the: (a) TaO($X^2\Delta$), (b) TaO($K^2\Phi$), (c) ZrS($X^1\Sigma^+$) and (d) ZrS($B^1\Pi$).

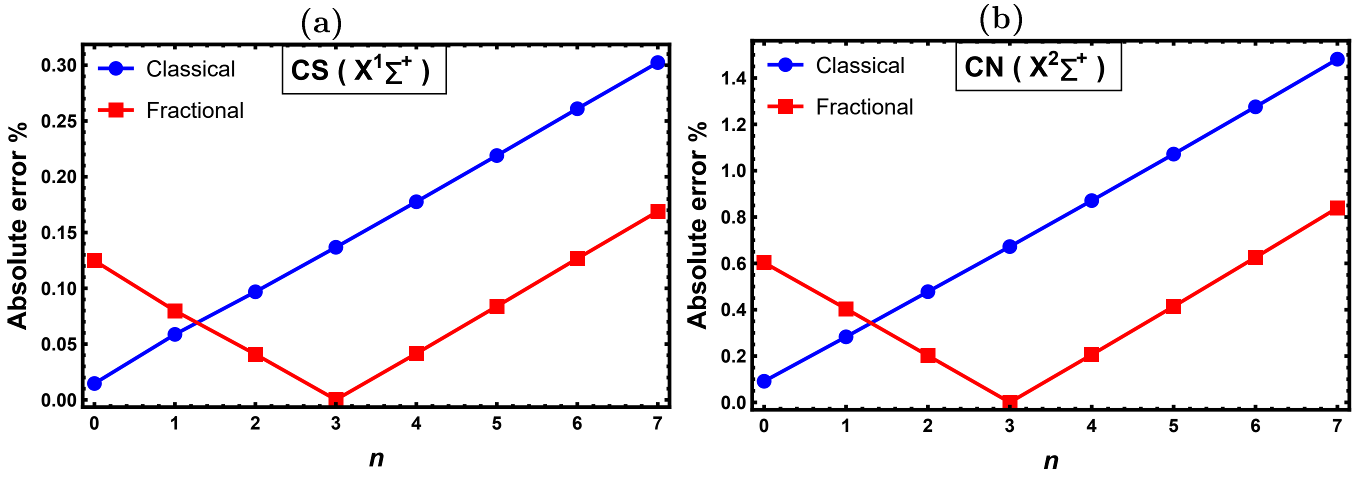

Fig. A6. Absolute percentage deviations of vibrational energies for the: (a) CS($X^1\Sigma^+$) and (b) CN($X^2\Sigma^+$).
